# Critical points and syzygies for Feynman integrals

**DOI:** 10.1007/JHEP02(2026)004

**Published:** 2026-02-02

**Authors:** Ben Page, Qian Song

**Affiliations:** Department of Physics and Astronomy, Ghent University, Proeftuinstraat 86, Ghent, Belgium

**Keywords:** Differential and Algebraic Geometry, Higgs Production, Scattering Amplitudes

## Abstract

We investigate a novel theoretical structure underlying the computation of integration-by-parts relations between Feynman integrals via syzygy-based methods. Building on insights from intersection theory, we analyze the large-*ϵ* limit of dimensional regularization on the maximal cut, showing that total derivatives vanish on the critical locus of the logarithm of the Baikov polynomial — the locus known to govern the number of master integrals. We introduce “critical syzygies” as a distinguished subset of syzygies that captures this behavior. We show that, when the critical locus is isolated, critical syzygies generate a sufficient set of total derivatives in the large-*ϵ* limit. We study their structure analytically at one loop and develop a numerical approach for their construction at two loops. Our results demonstrate that critical syzygies are a valuable tool for integral reduction in cutting-edge two-loop examples, offering a novel geometric perspective on integration-by-parts relations.

ArXiv ePrint: 2509.17681

## Contents

**1 Introduction** **1**

**2 Feynman integral relations in the Baikov representation** **3**

**3 Integral relations and critical points** **6**
- 3.1 Syzygies and geometry 6
- 3.2 From geometry to algebra 8
- 3.3 Syzygies and critical points 11
- 3.4 Critical surface terms 13
- 3.5 Multiplicity structure 15

**4 One-loop critical syzygies** **17**
- 4.1 Regular cases 18
- 4.2 Singular cases 19

**5 Critical surface terms at two loops** **22**
- 5.1 Computational construction of critical surface terms 23
- 5.2 Application to planar contributions to $pp \to t\bar{t}H$ at two loops 29

**6 Summary and outlook** **32**

**A Extended splitting lemma** **33**

## 1 Introduction

Feynman integrals are an essential component of precise predictions of scattering observables at particle colliders. Moreover, they are increasingly important for making predictions for gravitational wave experiments. In cutting-edge calculations in perturbative quantum field theory, one of the major bottlenecks is the large number of Feynman integrals that arise. For this reason, in modern computational frameworks, one makes extensive use of the fact that Feynman integrals exhibit linear relations with rational coefficients. These so-called "integration by parts" (IBP) relations [1, 2] are then used to reduce the number of integrals to a much smaller number of "master integrals". Moreover, these integral relations are essential for the calculation of Feynman integrals, forming the backbone of the "differential equations" approach [3–6].

Given the large importance of relations between Feynman integrals in perturbative calculations, it is perhaps not surprising that a large amount of effort has gone into understanding them. The classical approach to integral reduction, under the name of the "Laporta" algorithm [7], is to construct a large set of total derivatives, which integrate to zero. One then interprets these as relations between Feynman integrals and solves the relations using linear algebra. This method has been very successful, and many public implementations make use of it [8–15]. In recent years, many such approaches have been able to reach new heights by

JHEP02(2026)004

performing the reduction on numerical phase-space points using modular arithmetic [16–18] and reconstructing analytic results from these evaluations. In tandem, a number of approaches have been introduced to organize the set of total derivatives that one handles. One such approach is the "block-triangular form", where a system of total derivatives is constructed which allows for rapid numerical evaluations [14, 19]. Another approach recently under study, is to organize the relations to form symbolic reduction rules [20, 21]. Moreover, there have been a number of recent investigations into the application of machine-learning techniques to improving the Laporta algorithm [22–24]. Beyond this, there are the so-called "syzygy-based" approaches [25], which avoid introducing auxiliary terms into the collection of total derivatives that one constructs. A further related approach, recently under study is that of constructing parametric annihilators [26]. There have also been important developments in understanding mathematical structures that control the IBP relations, in the language of "intersection theory" [27, 28]. Here, one makes use of the "intersection product" that one can define on Feynman integrals in Baikov representation to directly reduce integrals to master integrals.

In this work, we study the "syzygy" approach, motivated by its importance in the two-loop numerical unitarity method [29–32]. Since its introduction in ref. [25], the syzygy approach to relations between Feynman integrals has received a great deal of study. Beyond its original formulation in momentum space, it has been formulated in so-called "adapted coordinates" [29, 33], embedding space [34–36], and, prominently, the Baikov representation [37, 38]. Many methods of computing solutions of the syzygy problem have been proposed. While some solutions are known in closed form [33, 39], most approaches to the syzygy problem are computational in nature. Early approaches made use of Groebner basis techniques based upon Schreyer's theorem [40, 41] as implemented in computer algebra packages such as Singular [42]. More recent approaches make use of the "module intersection" strategy [43] in Baikov representation. Another prominent approach is to reduce the syzygy problem to that of solving linear systems [44–47]. By now, the problem of syzygy construction is well enough understood that there exist public codes such as `NeatIBP` [48, 49].

In this work, we introduce a novel theoretical contribution to the understanding of syzygies and Feynman integrals. Specifically, we uncover a deep connection between these methods and recent applications of intersection theory [27, 50] to Feynman integrals. In recent work [51], it was observed that the intersection theory of dimensionally-regulated Feynman integrals greatly simplifies in the limit of large dimension — the large-$\epsilon$ limit. In the first part of our work, inspired by this observation, we study the large-$\epsilon$ limit of total derivatives in the syzygy formalism. In this limit, we observe that, when taken on the maximal cut, total derivatives vanish on the critical locus of the logarithm of the Baikov polynomial: exactly the locus studied in ref. [51]. We then formulate this observation in the language of algebraic geometry, discussing how this geometric statement arises from theoretical considerations of syzygies. Specifically, we highlight a connection between syzygies and the "ideal quotient": a geometrical operation which corresponds to the removal of branches of an algebraic variety. This theoretical correspondence allow us to define a distinguished subset of syzygies that we dub "critical syzygies" — those singled out in the large-$\epsilon$ limit. Appealing to the Lee-Pomeransky approach for counting master integrals [52], we then argue that, in cases where the critical locus of the maximal cut of the Baikov is isolated, they give rise to a complete collection of total derivatives in the large-$\epsilon$ limit.

In the rest of our work, we study this critical syzygy construction. We first discuss how critical syzygies arise in the context of one-loop Feynman integrals and how they are controlled by the geometry of the critical locus of the Baikov polynomial. In particular, we directly show how critical syzygies give rise to a complete set of total derivatives relevant for gauge theories in cases where the critical locus of the logarithm of the maximal-cut Baikov polynomial is isolated. We then turn to the treatment of critical syzygies at two loops, where their construction is mathematically much more complicated and we satisfy ourselves with computational studies. An important point is to understand how critical syzygies can be used to generate the complete set of total derivatives beyond the large-$\epsilon$ limit. To study this question, we develop a computational approach to the construction of critical syzygies. We then apply this approach to the cutting-edge two-loop $pp \to t\bar{t}H$ process. In this way, we are able to demonstrate that, in examples where the critical locus of the maximal-cut Baikov is isolated, critical syzygies are indeed sufficient to generate all necessary total derivatives.

The paper is organized as follows. In section 2 we introduce our setup of syzygies and surface terms in the Baikov representation. In section 3 we discuss the syzygy method in the large-$\epsilon$ limit and how this gives rise to the phenomenon of critical syzygies. In section 4 we discuss the analytic construction of critical syzygies for one-loop Feynman integrals and discuss where they do and do not generate a complete set of total derivatives. In section 5, we discuss critical syzygies at two loops and explore their completeness computationally in a series of examples focused on the two-loop five-point $pp \to t\bar{t}H$ process. Finally, in section 6, we summarize and discuss future directions.

## 2 Feynman integral relations in the Baikov representation

In this section, we introduce the key objects under study: Feynman integrals and their relations. We will organize these relations in the so-called "syzygy" formalism, particularly focusing on the construction of "surface terms", due to their importance in the numerical unitarity method. Let us consider a dimensionally-regulated, $l$-loop Feynman integral, that depends on $E$ independent external momenta $p_1, \ldots, p_E$. Each such Feynman integral can be associated to a graph, $\Gamma$. We will work in the Baikov representation [37]. We refer the reader to ref. [53] for a recent, detailed derivation of the representation. In the Baikov representation, a Feynman integral $I_\Gamma(\mathcal{N}, \vec{\nu})$ associated to a graph $\Gamma$, with numerator $\mathcal{N}$ and propagator powers $\vec{\nu}$ is given as

$$I_\Gamma(\mathcal{N}, \vec{\nu}) = \frac{c(D)}{G(p_1, \ldots, p_E)^{\gamma + l/2}} \int_{\mathscr{C}} \mathrm{d}^N \vec{z} \left[ B(\vec{z})^\gamma \frac{\mathcal{N}}{\prod_{e \in \mathrm{props}(\Gamma)} z_e^{\nu_e}} \right], \tag{2.1}$$

where $\gamma = (D - E - l - 1)/2$, $N = \binom{l}{2} + lE$ is the number of Baikov variables (correspondingly the number of independent scalar products in the diagram) and $c$ is an overall prefactor which depends only on the dimensional regulator. The function $G(p_1, \ldots, p_E)$ is the Gram determinant of the independent external momenta in the Feynman integral, given by

$$G(a_1, \ldots, a_m) = \det(a_i \cdot a_j). \tag{2.2}$$

The denominator product in eq. (2.1) is taken over the set of propagators associated to the graph $\Gamma$, which we collect into the set of indices, props$(\Gamma)$. We refer to the remaining set

JHEP02(2026)004

of Baikov variables as "irreducible scalar products", defining the associated set of indices ISPs(Γ) through

$$\{z_1, \dots, z_N\} = \{z_e \ : \ e \in \text{props}(\Gamma)\} \cup \{z_i \ : \ i \in \text{ISPs}(\Gamma)\}. \tag{2.3}$$

For notational convenience, as in eq. (2.3), we will often denote propagator or "edge" variables as $z_e$ and ISP variables as $z_i$. The function $B(\vec{z})$ is known as the "Baikov polynomial" and can be determined by expressing the Gram determinant of the loop momenta and external momenta in the Feynman integral in terms of Baikov variables. The integration contour, $\mathscr{C}$ in eq. (2.1), has a boundary given by the vanishing locus of the Baikov polynomial. That is,

$$\partial\mathscr{C} = \{\vec{z} \in \mathbb{R}^N \ : \ B(\vec{z}) = 0\}. \tag{2.4}$$

More explicit details of the contour will not be needed for our discussion.

An important fact about dimensionally-regulated Feynman integrals is that, in an appropriate representation, if the integrand is a total derivative, then the integral is zero. In the Baikov representation, this arises as

$$0 = \int_{\mathscr{C}} \mathrm{d}^N\vec{z}\, [\partial_k (B^\gamma f_k)] \,, \tag{2.5}$$

where the $k$ index is summed over all Baikov variables and the $f_k$ are rational functions of Baikov variables. A relation such as eq. (2.5) is known as an "integration-by-parts" or "IBP" relation. IBP relations follow as the total derivative integral can be re-written as an integral over the integration boundary, $\partial\mathscr{C}$. However, as the exponent $\gamma$ of the Bakov polynomial is taken generic, the integrand vanishes on the boundary and the result is zero. As the integrand of all Feynman integrals comes with a factor of $B^\gamma$, it is useful to rewrite eq. (2.5) as

$$0 = \int_{\mathscr{C}} \mathrm{d}^N\vec{z}\, [B^\gamma \nabla_k f_k] \,, \tag{2.6}$$

where we introduce the twisted covariant derivative $\vec{\nabla}$, which acts as

$$\nabla_k f_k = \partial_k f_k + \gamma f_k (\partial_k \log[B]). \tag{2.7}$$

If we consider appropriate choices of $\vec{f}$ in eq. (2.5) then we are led to the IBP relations for Feynman integrals. The space of Feynman integrands modulo these IBP relations is known as the space of master integrals.

A typical approach to the construction of relations between Feynman integrals is to judiciously construct $\vec{f}$ and to act on them with $\vec{\nabla}$. The resulting integrands then integrate to zero by eq. (2.6). These relations can then be organized by linear algebra methods, broadly known as the Laporta algorithm [7]. In ref. [33], it was observed that, when computing scattering amplitudes, it can be important to be able to construct total derivatives with a prescribed denominator structure. The content of the integral relations then is encoded in the numerators of the relations. This leads to the definition of the vector space of so-called "surface terms" as

$$\text{Surface}(\Gamma, \vec{\nu}) = \left\{ \mathcal{N} \ : \ \frac{\mathcal{N}}{\prod_{e\in\text{props}(\Gamma)} z_e^{\nu_e}} = \nabla_k \left[ \frac{a_k}{B^\Delta \prod_{e\in\text{props}(\Gamma)} z_e^{\beta_e}} \right] \right\}, \tag{2.8}$$

JHEP02(2026)004

where $\Delta$ and the $\beta_e$ are non-negative integers and we require that $\mathcal{N}$ and the $a_k$ belong to

$$R = \mathbb{C}(p_i \cdot p_j, m_k^2, \epsilon)[z_1, \dots, z_N]. \tag{2.9}$$

That is, the $a_k$ are polynomials in Baikov variables, but rational in scalar products of the external momenta, particle masses and the dimensional regulator $\epsilon$. The set of surface terms is therefore an infinite dimensional $\mathbb{C}(p_i \cdot p_j, m_k^2, \epsilon)$-subspace of the polynomial ring $R$. Understanding how to explicitly construct a basis of the subspace of $\text{Surface}(\Gamma, \vec{\nu})$ relevant for integral reduction is the main topic of this work.

In practice, controlling the integral relations via the Laporta algorithm, or constructing surface terms can prove demanding. To this end, an observation made in ref. [25] about Feynman integrals is that IBP relations can be controlled by studying "syzygies", which allow one to directly generate linear relations between Feynman integrals that do not have raised propagator powers. We will work with syzygies in the Baikov representation, originally studied in ref. [38] and will consider syzygies of the form

$$0 = a_0 B + \sum_{i \in \text{ISPs}(\Gamma)} a_i \partial_i B + \sum_{e \in \text{props}(\Gamma)} \tilde{a}_e z_e B + \sum_{e \in \text{props}(\Gamma)} \overline{a}_e z_e \partial_e B, \tag{2.10}$$

where the $a_0, a_i, \tilde{a}_e, \overline{a}_e$ are members of $R$. That is, we are looking for tuples $a_0, a_i, \tilde{a}_e, \overline{a}_e$ of polynomials in Baikov variables, whose coefficients are rational functions in the external kinematics and $\epsilon$, such that eq. (2.10) is satisfied. We will denote the set of solutions to eq. (2.10) as $\text{Syz}(\Gamma)$. Importantly, the elements of $\text{Syz}(\Gamma)$ form an $R$-module. That is, taking $R$-linear combinations of solutions of eq. (2.10) yields other solutions. We note that the syzygy relation eq. (2.10) is subtly different to the one used in ref. [38], due to the $\tilde{a}_e$ term. In practice, it has the effect that the $a_0$ term can be studied on the maximal cut, as all terms proportional to propagators can be moved into the $\tilde{a}_e$. This slight adjustment to the formalism of ref. [38] will turn out to be fruitful later.

Let us consider how eq. (2.10) aids in the construction of integral relations. We note that eq. (2.10) is a zero at the level of polynomials, i.e. it is an integrand relation. However, its form allows one to easily apply integration by parts in order to end up with an interesting relation that is between integrals. Specifically, by pre-multiplying eq. (2.10) with $\frac{B^{\gamma-1}}{\prod_{e \in \text{props}(\Gamma)} z_e^{\nu_e}}$, we can rewrite it as

$$0 = \frac{1}{\prod_{e \in \text{props}(\Gamma)} z_e^{\nu_e}} \left[ a_0 B^\gamma + \frac{1}{\gamma} \sum_{i \in \text{ISPs}(\Gamma)} a_i \partial_i [B^\gamma] + \sum_{e \in \text{props}(\Gamma)} \left( \tilde{a}_e z_e B^\gamma + \frac{1}{\gamma} \overline{a}_e z_e \partial_e [B^\gamma] \right) \right]. \tag{2.11}$$

If we now integrate this 0 over $\mathscr{C}$, we can perform partial integrations on the $a_i$ and $\overline{a}_e$ terms to find a relation between Feynman integrals,

$$0 = \int_{\mathscr{C}} \mathrm{d}^N \vec{z} \left[ B^\gamma \frac{S_\Gamma(\vec{a}, \vec{\nu})}{\prod_{e \in \text{props}(\Gamma)} z_e^{\nu_e}} \right], \tag{2.12}$$

where we define

$$S_\Gamma(\vec{a}, \vec{\nu}) = a_0 + \sum_{e \in \text{props}(\Gamma)} \tilde{a}_e z_e - \frac{1}{\gamma} \left[ \sum_{i \in \text{ISPs}(\Gamma)} \partial_i a_i + \sum_{e \in \text{props}(\Gamma)} (z_e \partial_e \overline{a}_e - (\nu_e - 1) \overline{a}_e) \right]. \tag{2.13}$$

JHEP02(2026)004

In this way, we see that a syzygy of the form (2.10) induces a relation between Feynman integrals of the same dimension and without raising propagator powers. It is clear that the $S_\Gamma(\vec{a},\vec{\nu})$ of eq. (2.12) is an element of $\text{Surface}(\Gamma,\vec{\nu})$, and in fact $S_\Gamma$ maps the set of syzygyies to the set of surface terms. This leads us to define

$$\text{SyzSurface}(\Gamma,\vec{\nu}) = \{S_\Gamma(\vec{a},\vec{\nu}) \;:\; \vec{a} \in \text{Syz}(\Gamma)\}\,, \tag{2.14}$$

the set of surface terms constructed from syzygies, a manifest subspace of $\text{Surface}(\Gamma,\vec{\nu})$. It is important to observe that there is no claim that the space of surface terms arising from syzygies is the full space of surface terms. Indeed, experience tells us that this is generally not the case. One of the contributions of this work is to develop a criteria for when we can expect the two spaces to be equal.

## 3 Integral relations and critical points

When working in the syzygy formalism introduced in the previous section, one is faced with the natural question of how to construct the set of syzygies, $\text{Syz}(\Gamma)$. In practice, this turns out to be a difficult problem. In this work, we make progress on this problem by using geometrical methods to identify a subset of $\text{Syz}(\Gamma)$ which, in a broad set of cases, generate a sufficient set of surface terms for reduction to master integrals. To begin, we decide to consider the surface term in eq. (2.13) for large values of the dimensional regularization parameter, while also dropping terms that vanish on the maximal cut. The perhaps surprising decision to consider the "large-$\epsilon$ limit" is motivated by the success of this strategy in the context of intersection theory, where taking this limit induces important simplifications [50, 51]. In such a regime, we find that the surface term reduces to simply

$$\left[\lim_{\epsilon\to\infty} S_\Gamma(\vec{a},\vec{\nu})\right]\Big|_{\text{cut}_\Gamma} = a_0|_{\text{cut}_\Gamma}, \tag{3.1}$$

where by $f|_{\text{cut}_\Gamma}$, we mean that we evaluate $f$ on $z_e = 0$ for $e \in \text{props}(\Gamma)$, i.e. we evaluate $f$ on the maximal cut of $\Gamma$. We therefore see that, in this regime, we need to only consider the $a_0$ term on the maximal cut. This represents a dramatic simplification of the IBP relation, as only a single term from the syzygy relation contributes. This observation provides a strong motivation to study the $a_0$ term of eq. (2.10) alone. In this section, we shall study this piece geometrically and interpret these features in the language of algebraic geometry. For background on this language, we direct the reader to standard textbooks such as [54].

### 3.1 Syzygies and geometry

Our aim is to understand the unknown polynomial $a_0$ in eq. (2.10) by considering it geometrically. In principle, the techniques introduced here can be used to study other terms, but we leave such investigations to further work. In order to isolate the $a_0$ term in the syzygy, it is natural to consider setting all of the other terms in eq. (2.10) to zero. To this end, we consider setting

$$\begin{aligned} \partial_i B = 0, \qquad & i \in \text{ISPs}(\Gamma), \\ z_e B = z_e \partial_e B = 0, \qquad & e \in \text{props}(\Gamma). \end{aligned} \tag{3.2}$$

JHEP02(2026)004

If we consider these equations as constraints on the $\vec{z}$ variables, we see that, for fixed external kinematics, they cut out a surface in $\mathbb{C}^N$. As the equations in eq. (3.2) are algebraic, this surface is an algebraic variety. We will refer to this variety as the "syzygy" variety associated to $\Gamma$, which we will denote as $U^{\Gamma}_{\text{syz}}$. Importantly, for any point $\vec{z}$ on the syzygy variety, eq. (2.10) reduces to

$$a_0 B|_{U^{\Gamma}_{\text{syz}}} = 0, \tag{3.3}$$

and we see that we have successfully isolated the $a_0$ term in the syzygy. We therefore see that $U^{\Gamma}_{\text{syz}}$ is of prime importance, so we consider its structure. Given that a number of the defining equations in eq. (3.2) factorize, the syzygy variety can naturally be decomposed into subvarieties.[1] A first observation is that it naturally splits into two subvarieties where $B = 0$ and $B \neq 0$. That is,

$$U^{\Gamma}_{\text{syz}} = U^{\subseteq\Gamma}_{\text{sing}} \cup U^{\Gamma}_{\text{crit}[\log(B)]}. \tag{3.4}$$

The first variety, $U^{\subseteq\Gamma}_{\text{sing}}$, corresponds to the case $B = 0$. By consideration of eq. (3.2) we see that it is composed of a large number of subvarieties where either propagators are cut, or derivatives of the Baikov polynomial are set to zero. That is, to each set of edges $\Gamma_k \subseteq \Gamma$ we consider $U^{\Gamma_k}_{\text{sing}}$, the variety in $\mathbb{C}^N$ defined by

$$\begin{aligned} \partial_i B &= 0 \;:\; i \in \text{ISPs}(\Gamma_k), \\ z_e &= 0 \;:\; e \in \text{props}(\Gamma_k), \\ B &= 0. \end{aligned} \tag{3.5}$$

Geometrically, each $U^{\Gamma_k}_{\text{sing}}$ corresponds to the singular locus of the Baikov polynomial on the cut $\Gamma_k$, that is, where the surface fails to be smooth. Explicitly, $U^{\subseteq\Gamma}_{\text{sing}}$ is the union of all these varieties, i.e.

$$U^{\subseteq\Gamma}_{\text{sing}} = \bigcup_{\Gamma_k \subseteq \Gamma} U^{\Gamma_k}_{\text{sing}}. \tag{3.6}$$

The second variety, $U^{\Gamma}_{\text{crit}[\log(B)]}$ corresponds to the case where $B \neq 0$. Looking once again at eq. (3.2), we see that if the Baikov polynomial is non-zero, then the propagators in $\Gamma$ must be zero and hence $U^{\Gamma}_{\text{crit}[\log(B)]}$ is defined by the equations

$$\begin{aligned} \partial_i \log(B) &= 0 \;:\; i \in \text{ISPs}(\Gamma), \\ z_e &= 0 \;:\; e \in \text{props}(\Gamma), \end{aligned} \tag{3.7}$$

where we make use of the logarithm to enforce that $B \neq 0$. Geometrically, we see that $U^{\Gamma}_{\text{crit}[\log(B)]}$ is the locus where the logarithm of the $\Gamma$-cut Baikov polynomial reaches its extremal, or "critical" values.

Having understood the syzygy variety itself, let us consider what it tells us about $a_0$. Considering eq. (3.3), in order to further isolate the $a_0$ term, we impose that the factor

[1] In practical explorations, one also finds that there are further decompositions that are not manifest in eq. (3.2). We leave systematic understanding of these branchings to further work.

JHEP02(2026)004

of $B$ does not vanish. That is, we consider eq. (3.3) restricted to $U^{\Gamma}_{\mathrm{crit}[\log(B)]}$ and find that $a_0$ must vanish there, i.e.

$$a_0|_{U^{\Gamma}_{\mathrm{crit}[\log(B)]}} = 0. \tag{3.8}$$

In words, we see that $a_0$ vanishes on the critical locus of the logarithm of the Baikov polynomial, on the cut corresponding to the graph $\Gamma$. This observation is of strong importance, as this process-independent constraint on a piece of the syzygy connects the syzygy formalism to other recent advances in the understanding of relations between Feynman integrals. Specifically, both the Lee-Pomeransky approach to counting master integrals [52] as well as recent advances in the application of intersection theory to Feynman integrals [51] make use of the variety $U^{\Gamma}_{\mathrm{crit}[\log(B)]}$. It is well-known that in many cases this variety is a finite set of points and Lee and Pomeransky showed in ref. [52] that, in such cases, the number of these points, when counted with multiplicity, is exactly the number of master integrals associated to the topology $\Gamma$. Moreover, this statement is reinforced in the intersection theory literature, where intersection numbers can be written in terms of evaluations of the integrand on the points of $U^{\Gamma}_{\mathrm{crit}[\log(B)]}$. It is therefore perhaps not surprising that syzygies of Feynman integrals would also exhibit a connection to $U^{\Gamma}_{\mathrm{crit}[\log(B)]}$. In this work, we shall explore how to constructively use this connection to build syzygies for Feynman integrals.

### 3.2 From geometry to algebra

In the previous subsection, we have gained a geometric insight into a piece of the syzygy relation (2.10), demonstrating a connection to the variety $U^{\Gamma}_{\mathrm{crit}[\log(B)]}$. However, the connection currently remains unconstructive. While eq. (3.8) tells us that all $a_0$ must vanish on the critical locus of $\log(B)$ on the cut associated to $\Gamma$, it is unclear if this property is sufficient or simply necessary. Indeed, when considering eq. (3.3) closely, we notice a potential subtlety: we are unable to exclude the possibility that $a_0$ must also vanish on $U^{\subsetneq\Gamma}_{\mathrm{sing}}$. In order to gain control of this subtlety, we shall rephrase our geometric discussion in the algebraic language of ideals. Let us consider the terms of eq. (2.10) other than $a_0 B$. By inspection, we see that they parameterize an element of the ideal

$$J^{\Gamma}_{\mathrm{syz}} = \langle \partial_i B \; : \; i \in \mathrm{ISPs}(\Gamma) \rangle + \langle z_e B, z_e \partial_e B \; : \; e \in \mathrm{props}(\Gamma) \rangle. \tag{3.9}$$

Here we denote the ideal generated by $\{g_1, \ldots\}$ as $\langle g_1, \ldots \rangle$ and $+$ denotes the ideal sum. The ideal $J^{\Gamma}_{\mathrm{syz}}$ is an ideal of the polynomial ring $R$ defined in eq. (2.9): polynomials in Baikov variables, with coefficients that are rational functions of external kinematics and $\epsilon$. Importantly, the syzygy variety that we identified earlier, $U^{\Gamma}_{\mathrm{syz}}$ is the variety associated to the ideal $J^{\Gamma}_{\mathrm{syz}}$. That is,[2]

$$U^{\Gamma}_{\mathrm{syz}} = V(J^{\Gamma}_{\mathrm{syz}}). \tag{3.10}$$

The importance of $J^{\Gamma}_{\mathrm{syz}}$ is that it encodes algebraic features of the syzygies, such as multiplicity, which we can associate to the variety $U^{\Gamma}_{\mathrm{syz}}$. Having seen the importance of $U^{\Gamma}_{\mathrm{syz}}$, let us similarly

[2]We recall that the variety $V(J)$ associated to an ideal $J$ of $\mathbb{F}[z_1, \ldots, z_N]$, for some field $\mathbb{F}$, is the set of $\vec{z} \in \mathbb{F}^N$ such that $p(\vec{z}) = 0$ for all $p \in J$ and refer the reader to ref. [54] for more details.

JHEP02(2026)004

analyze $J^{\Gamma}_{\text{syz}}$. Similar to the splitting of the associated variety, it is possible to prove an analogous splitting for $J^{\Gamma}_{\text{syz}}$. In contrast to splitting a variety, which expresses it as the union of multiple subvarieties, an ideal can be expressed as the intersection of other ideals, each of which is larger than the initial ideal. A particularly relevant splitting of $J^{\Gamma}_{\text{syz}}$ is induced by the explicit factors of $B$ in some of its generators. In order to perform this splitting, we make use of the lemma proven in appendix A and write $J^{\Gamma}_{\text{syz}}$ as

$$J^{\Gamma}_{\text{syz}} = J^{\subseteq\Gamma,\mu}_{\text{sing}} \cap J^{\Gamma}_{\text{crit}(B)}, \tag{3.11}$$

where we define

$$J^{\subseteq\Gamma,\mu}_{\text{sing}} = J^{\Gamma}_{\text{syz}} + \langle B^{\mu}\rangle, \tag{3.12}$$

$$J^{\Gamma}_{\text{crit}(B)} = \langle \partial_i B : i \in \text{ISPs}(\Gamma)\rangle + \langle z_e : e \in \text{props}(\Gamma)\rangle. \tag{3.13}$$

The two ideals get their names from their associated varieties as $V(J^{\subseteq\Gamma,\mu}_{\text{sing}}) = U^{\subseteq\Gamma}_{\text{sing}}$ and $V(J^{\Gamma}_{\text{crit}(B)})$ is the critical locus of the Baikov polynomial on the cut associated to $\Gamma$. The exponent $\mu$ in eq. (3.12) is a positive integer known as the "saturation index" of $J^{\Gamma}_{\text{syz}}$ with respect to the Baikov polynomial. It represents the multiplicity of the $B = 0$ component of $J^{\Gamma}_{\text{syz}}$, and its value is *a priori* unknown. Practical experience says that it is often 1, but there exist physical examples, such as that discussed in section 5.2, where it is higher.

Having introduced $J^{\Gamma}_{\text{syz}}$ let us now consider how we can use it to understand the $a_0$ term. From eq. (2.10) we see that $a_0$ is any polynomial in $R$, such that when you multiply it by the Baikov polynomial, you get an element of $J^{\Gamma}_{\text{syz}}$. This leads us to define the set of all possible $a_0$ terms as

$$A^{\Gamma}_0 = \left\{ p \in R \ : \ pB \in J^{\Gamma}_{\text{syz}} \right\}. \tag{3.14}$$

Importantly, it is not hard to see that $A^{\Gamma}_0$ is also an ideal of $R$. By consideration of eq. (3.1) we are therefore able to make the remarkable statement that on the maximal cut and in the large-$\epsilon$ limit, surface terms actually have the structure of an ideal. This observation will allow us to better understand the structure of surface terms using methods from the theory of ideals. In general, explicitly finding a generating set for $A^{\Gamma}_0$ is a non-trivial task. For the moment, we content ourselves with making structural statements.

We begin by observing that the set in eq. (3.14) is actually the definition of the "ideal quotient" of $J^{\Gamma}_{\text{syz}}$ by the ideal generated by the Baikov polynomial. That is, we have that

$$A^{\Gamma}_0 = J^{\Gamma}_{\text{syz}} : \langle B\rangle. \tag{3.15}$$

The relation in eq. (3.15) is the crucial constructive observation of this work, allowing us to study the problem of determining syzygies with the technology of ideal quotients. A first important property of ideal quotients is that they have a geometrical significance. Specifically, the ideal quotient can be used to implement the set difference of two varieties: to remove one variety from another. The relation relevant to our construction is

$$V[J^{\Gamma}_{\text{syz}} : \langle B^{\mu}\rangle] = \overline{V[J^{\Gamma}_{\text{syz}}] \setminus V(\langle B\rangle)} = U^{\Gamma}_{\text{crit}[\log(B)]}, \tag{3.16}$$

JHEP02(2026)004

where the bar represents that we take the Zariski closure.[3] That is, if one quotients $J^{\Gamma}_{\text{syz}}$ by $B^{\mu}$, the associated variety is $U^{\Gamma}_{\text{syz}}$ with the $B = 0$ component removed. From the previous section, we see that this is just $U^{\Gamma}_{\text{crit}[\log(B)]}$. Importantly, eq. (3.16) gives us a geometrical interpretation of the saturation index $\mu$. As $\mu$ represents the multiplicity of the $B = 0$ component of $J^{\Gamma}_{\text{syz}}$, removing it requires quotienting $J^{\Gamma}_{\text{syz}}$ by $B$ "$\mu$ times". A second important property of ideal quotients is that they act on each component of an intersection.[4] By consideration of eq. (3.11), this allows us to write $A^{\Gamma}_0$ as an intersection of ideals as

$$A^{\Gamma}_0 = \left(J^{\Gamma}_{\text{crit}(B)} : \langle B \rangle\right) \cap \left(J^{\subseteq\Gamma,\mu}_{\text{sing}} : \langle B \rangle\right). \tag{3.17}$$

This relation allows us to robustly understand the connection of $A^{\Gamma}_0$ to our geometrical considerations. First, let us consider the set of all polynomials that vanish on $U^{\Gamma}_{\text{crit}[\log(B)]}$, denoted $I(U^{\Gamma}_{\text{crit}[\log(B)]})$. By applying Hilbert's strong Nullstellensatz to eq. (3.16), we have that

$$I(U^{\Gamma}_{\text{crit}[\log(B)]}) = \sqrt{J^{\Gamma}_{\text{crit}(B)} : \langle B^{\mu} \rangle}, \tag{3.18}$$

where the square root of an ideal denotes taking its radical. Using elementary properties of radicals, quotients and intersections, it is not difficult to conclude that

$$A^{\Gamma}_0 \subseteq I(U^{\Gamma}_{\text{crit}[\log(B)]}). \tag{3.19}$$

That is, in general, $A^{\Gamma}_0$ is a subset of the polynomials which vanish on $U^{\Gamma}_{\text{crit}[\log(B)]}$. In other words, we see that while $a_0$ must vanish on $U^{\Gamma}_{\text{crit}[\log(B)]}$, it may also satisfy further non-trivial constraints.

In practice, it turns out that there is an important case where eq. (3.17) can be shown to simplify: where $\mu = 1$. As we will discuss in sections 4 and 5, we experimentally find that this is almost always the case for Feynman integrals. Let us, therefore, analyze the $\mu = 1$ case in detail. In this case, if we look to the definition of $J^{\subseteq\Gamma,\mu}_{\text{sing}}$ in eq. (3.12), we see that any polynomial in $R$ multiplied by the Baikov polynomial is in $J^{\subseteq\Gamma,\mu}_{\text{sing}}$, as the Baikov polynomial is a generator. Hence, we see that the right intersectand of eq. (3.17) is $R$ and as $R$ is the identity under intersection, we conclude that

$$\mu = 1 \qquad \Rightarrow \qquad A^{\Gamma}_0 = J^{\Gamma}_{\text{crit}(B)} : \langle B \rangle. \tag{3.20}$$

That is, our ideal quotient simplifies exactly if $\mu = 1$. By the definition of the saturation index, we see that, in this case, all $a_0$ terms must vanish on $U^{\Gamma}_{\text{crit}[\log(B)]}$. An important question that we will study experimentally in this work is what value of $\mu$ we will typically encounter. In practice, we will find that it is most often 1. Nevertheless, we will return to the question of how to interpret cases where $\mu \neq 1$ in section 3.5.

To close this section, let us note that there is a simple case where we can easily find a generating set for $A^{\Gamma}_0$. It follows by constructing a simple upper and lower bound for $A^{\Gamma}_0$. Specifically, one has that

$$J^{\Gamma}_{\text{crit}(B)} \subseteq A^{\Gamma}_0 \subseteq J^{\Gamma}_{\text{crit}(B)} : \langle B \rangle. \tag{3.21}$$

[3]The Zariski closure of a set is the smallest algebraic variety that contains that set. In the context of ideal quotients, this has the effect of filling in "holes" in the variety. Further details will not be required for our discussion.

[4]That is, for $R$-ideals $A$, $B$ and $C$, one has that $(A \cap B) : C = (A : C) \cap (B : C)$.

JHEP02(2026)004

The left inclusion follows by inspection of the generators of $J^\Gamma_{\mathrm{crit}(B)}$. Each of them, when multiplied by the Baikov polynomial, is an element of $J^\Gamma_{\mathrm{syz}}$, and hence the left inclusion follows. The right inclusion follows by consideration of eq. (3.17). Now, let us consider the case that $J^\Gamma_{\mathrm{crit}(B)} = J^\Gamma_{\mathrm{crit}(B)} : \langle B \rangle$. Then we have that the upper and lower bounds of eq. (3.21) are equal leading to $A^\Gamma_0 = J^\Gamma_{\mathrm{crit}(B)}$. In such a case, as a generating set of $J^\Gamma_{\mathrm{crit}(B)}$ is given, then we find a generating set for $A^\Gamma_0$. Interpreting this geometrically, we see that this corresponds to the case where $U^\Gamma_{\mathrm{sing}}$ is empty, i.e. the zero-set of the maximal cut Baikov polynomial is a smooth variety.

### 3.3 Syzygies and critical points

Having understood the $a_0$ term in the syzygy relation eq. (2.10) both geometrically and algebraically, we will now argue that the $a_0$ term is of deep importance to integration-by-parts relations. To this end, we recall the approach of Lee and Pomeransky in ref. [52] for counting the number of master integrals associated to a Feynman integral with graph $\Gamma$. To begin, let us identify the space of master integrals on the maximal cut of $\Gamma$ as the linearly independent numerators modulo surface terms and terms that vanish on the cut. That is,

$$H_\Gamma = R/(\mathrm{Surface}(\Gamma, \vec{1}) + J^\Gamma_{\mathrm{cut}}), \tag{3.22}$$

where we make use of

$$J^\Gamma_{\mathrm{cut}} = \langle z_e : e \in \mathrm{props}(\Gamma) \rangle \tag{3.23}$$

and by $\vec{1}$ we mean that the entries of the exponent vector $\vec{\nu}$ are all 1. The question of counting the number of master integrals can then be understood as counting the dimension of the vector space $H_\Gamma$. The approach of Lee and Pomeransky argues that $\dim(H_\Gamma)$ is encoded in the solution set of the equations

$$\begin{aligned} \partial_i B = 0 \ &: \ i \in \mathrm{ISPs}(\Gamma), \\ z_e = 0 \ &: \ e \in \mathrm{props}(\Gamma), \\ B \neq 0. & \end{aligned} \tag{3.24}$$

Precisely, in the case that the solution set of eq. (3.24) is a finite number of points, then the number of master integrals, $\dim(H_\Gamma)$, is equal to the number of solutions, counted with multiplicity. As alluded to earlier, note that eq. (3.24) is entirely equivalent to eq. (3.7) and so the Lee and Pomeransky approach is counting points in $U^\Gamma_{\mathrm{crit}[\log(B)]}$.

In ref. [52], in order to count the number of solutions to eq. (3.24) without directly computing the set of solutions, the authors introduce an "algebraic formulation", which is implemented in the code `MINT`. This formulation makes use of the ideal

$$J^\Gamma_{\mathrm{LP}} = \langle \partial_i B \ : \ i \in \mathrm{ISPs}(\Gamma), \quad z_e \ : \ e \in \mathrm{props}(\Gamma), 1 - wB \rangle_{R[w]} \cap R, \tag{3.25}$$

where $w$ is an auxiliary variable. The explicit ideal on the right-hand-side of eq. (3.25) is an ideal in the ring $R[w]$, the ring of both Baikov variables and $w$. The intersection with the ring $R$ eliminates the variable $w$, and can be implemented with Groebner basis

JHEP02(2026)004

techniques. The ideal $J^\Gamma_{\rm LP}$ can be used to compute the number of points in $V(J^\Gamma_{\rm LP})$ by exploiting special properties of an ideal $J$, whose associated variety $V(J)$ is a finite set of points. Specifically, the number of points, in $V(J)$ counted with multiplicity, is equal to the number of linearly independent polynomials when they are considered modulo the ideal $J$ [40, Chapter 4, Corollary 2.6]. That is, one takes the ring of polynomials $R$ and considers any two elements or $R$ that differ by an element of $J$ to be equivalent. Denoting this equivalence class ring as $R/J$, in the case where $V(J)$ is a finite set of points, it turns out that $R/J$ is a finite-dimensional vector space. The dimension of this vector space, $\text{vdim}(R/J)$ gives the desired point counting. Applying this to the ideal $J^\Gamma_{\rm LP}$ we arrive at the Lee-Pomeransky formula for counting master integrals,

$$\dim(H_\Gamma) = \text{vdim}\left(R/J^\Gamma_{\rm LP}\right). \tag{3.26}$$

Importantly, $\text{vdim}\left(R/J^\Gamma_{\rm LP}\right)$ can be computed without computing the solutions to eq. (3.24). It requires only a Groebner basis of $J^\Gamma_{\rm LP}$, which can easily be computed in modern computer algebra systems.

Naturally, the master integral counting of Lee and Pomeransky must in some way be connected to the set of syzygies, as they are intimately related to the construction of the surface terms. Nevertheless, it turns out that this connection can be made much more directly. Let us return to consider eq. (3.25). An important observation is that the ideal in eq. (3.25) can be identified as an application of the so-called "Rabinowitsch trick" to perform the ideal saturation of $J^\Gamma_{\text{crit}(B)}$ with respect to $B$ (see ref. [54, Chapter 4, Section 4, Theorem 14 (ii)]). That is, we have that

$$J^\Gamma_{\rm LP} = J^\Gamma_{\text{crit}(B)} : \langle B^\mu \rangle, \tag{3.27}$$

where $\mu$ is the saturation index of $J^\Gamma_{\text{crit}(B)}$ with respect to the Baikov polynomial. Therefore, in the case $\mu = 1$, we have that the Lee-Pomeransky ideal is exactly the ideal to which all $a_0$ terms must belong. That is

$$\mu = 1 \qquad \Rightarrow \qquad J^\Gamma_{\rm LP} = A^\Gamma_0 \tag{3.28}$$

This is a striking statement: the ideal involved in counting master integrals with the Lee-Pomeransky approach also arises in the syzygy approach.

The observation of the direct connection between the syzygy approach and the Lee-Pomeransky approach has important consequences for considering total derivatives. To see this, consider that $H_\Gamma$ and $R/J^\Gamma_{\rm LP}$ are two different quotient spaces of $R$ and as such they each furnish a decomposition of $R$ as a vector space. We can therefore write

$$\sigma_1(H_\Gamma) + \text{Surface}(\Gamma, \vec{1}) + J^\Gamma_{\rm cut} = \sigma_2(R/J^\Gamma_{\rm LP}) + J^\Gamma_{\rm LP}, \tag{3.29}$$

where the $\sigma_i$ are canonical maps that identify the quotient spaces as subspaces of $R$. That is, both the left- and right-hand side of eq. (3.29) can be recognized as a decomposition of $R$ into $R/W + W$ for some subspace $W$ and are therefore equal. Let us now assume that there exists a set of master integrals that are linearly independent on the points of $V(J^\Gamma_{\rm LP})$, i.e. on $U_{\text{crit}[\log(B)]}$. This is the statement that we can choose the $\sigma_i$ such that $\sigma_1(H_\Gamma) = \sigma_2(R/J^\Gamma_{\rm LP})$.

JHEP02(2026)004

In experience of practical applications to Feynman integrals, this is found to be true so we assume this from now on. Under this assumption, it is a simple application of a standard fact of linear algebra that the two spaces complementary to the $\sigma_i$ spaces in eq. (3.29) are isomorphic. Under the condition that $\mu = 1$ and $U^{\Gamma}_{\text{crit}[\log(B)]}$ is a set of points, we are therefore able to conclude that

$$A_0^{\Gamma} \simeq \text{Surface}(\Gamma, \vec{1}) + J^{\Gamma}_{\text{cut}}. \tag{3.30}$$

This equation, eq. (3.30), is an important result of our work. We interpret eq. (3.30) to say that a basis of $A_0^{\Gamma}$ is in one-to-one correspondence with a basis of surface terms, up to terms that vanish on the cut associated to $\Gamma$.

### 3.4 Critical surface terms

The correspondence between the space of $a_0$ parts of syzygies and surface terms that we have just identified suggests an interesting perspective on the syzygy formalism for surface term construction. Specifically, under the conditions that eq. (3.30) holds, we should only need a set of syzygies whose $a_0$ piece is linearly independent on the maximal cut, in order to be able to reduce to master integrals, modulo pinch integrals. This is a highly compelling observation as there are a large number of elements of $\text{Syz}(\Gamma)$ that satisfy $a_0 = 0$, but eq. (3.30) tells us that we can neglect them. Motivated by this, we will now define the set of "critical surface terms": those with linearly independent $a_0$ piece on the maximal cut.

To begin, let us define the "critical part" of an element $\vec{a}$ of $\text{Syz}(\Gamma)$ as the $a_0$ part, i.e.

$$\mathfrak{c}(\vec{a}) \coloneqq a_0. \tag{3.31}$$

Our discussion states that two syzygies that have the same critical part give the same on-shell relation between Feynman integrals in the large-$\epsilon$ limit. Beyond this, the introduction of the $\tilde{a}_e$ in eq. (2.10) terms means that two syzygies will give rise to the same surface term if they differ by an $R$-linear combination of syzygies of the form

$$a_0 = z_e, \qquad \tilde{a}_e = -1, \tag{3.32}$$

with all other entries being 0. We will denote the submodule of $\text{Syz}(\Gamma)$ generated by the syzygies of eq. (3.32) as $\text{ZSyz}(\Gamma)$ as they give rise to surface terms which are zero. Together, this leads us to observe that there is a natural equivalence relation on $\text{Syz}(\Gamma)$: two elements that differ either by some $\vec{w}_1 \in \text{ZSyz}(\Gamma)$ or $\vec{w}_2 \in \text{Syz}(\Gamma)$ such that $\mathfrak{c}(\vec{w}_2) = 0$ should be regarded as equivalent. Denoting this equivalence relation as $\sim$, we have

$$\vec{a} \;\sim\; \vec{a} + \vec{w}_1 + \vec{w}_2, \qquad \text{where} \quad \vec{a} \in \text{Syz}(\Gamma), \quad \vec{w}_1 \in \text{ZSyz}(\Gamma) \quad \text{and} \quad \vec{w}_2 \in \ker(\mathfrak{c}). \tag{3.33}$$

This motivates us to define the module of "critical syzygies" as the quotient of the module of syzygies by this equivalence relation. That is, we define

$$\text{CSyz}(\Gamma) = \text{Syz}(\Gamma)/(\ker(\mathfrak{c}) + \text{ZSyz}(\Gamma)), \tag{3.34}$$

where we recognize the set of elements that are equivalent to zero under $\sim$ as the module sum of the kernel of $\mathfrak{c}$ and $\text{ZSyz}(\Gamma)$. Elements of $\text{CSyz}(\Gamma)$ are equivalence classes under $\sim$,

JHEP02(2026)004

and can be represented by elements of $\mathrm{Syz}(\Gamma)$. For any element $\vec{a}$ of $\mathrm{Syz}(\Gamma)$, we denote the associated equivalence class in $\mathrm{CSyz}(\Gamma)$ as $[\vec{a}]$. Conversely, given $[\vec{a}] \in \mathrm{CSyz}(\Gamma)$, we will refer to a (non-unique) representative $\vec{a} \in \mathrm{Syz}(\Gamma)$ as a lift of $[\vec{a}]$. An important observation about $\mathrm{CSyz}(\Gamma)$ is its module structure. By construction, two elements of $\mathrm{CSyz}(\Gamma)$ are inequivalent only if their critical parts on the maximal cut are distinct. We therefore see that

$$\mathrm{CSyz}(\Gamma) \simeq A_0^\Gamma / J_{\mathrm{cut}}^\Gamma. \tag{3.35}$$

It is therefore clear that the set of critical syzygies is much "smaller" than the full set of syzygies, as it is only a rank 1 module.

Let us now consider using $\mathrm{CSyz}(\Gamma)$ to construct surface terms. A technicality here is that elements of $\mathrm{CSyz}(\Gamma)$ are equivalence classes of syzygies. That is, $\mathrm{CSyz}(\Gamma)$ is a quotient space of $\mathrm{Syz}(\Gamma)$. Nevertheless, as a quotient space of $\mathrm{Syz}(\Gamma)$, $\mathrm{CSyz}(\Gamma)$ is isomorphic to some subspace of $\mathrm{Syz}(\Gamma)$. That is, analogous to the $\sigma_i$ of eq. (3.29), there exists a linear map $\pi$ such that

$$\pi(\mathrm{CSyz}(\Gamma)) \subset \mathrm{Syz}(\Gamma). \tag{3.36}$$

Practically, defining $\pi$ can be thought of as finding a lift $\vec{a}_i$ for each basis vector $[\vec{a}_i]$ of $\mathrm{CSyz}(\Gamma)$. Naturally, the non-uniqueness of this lift implies that $\pi$ is not unique. Nevertheless, this map $\pi$ allows us to define a subspace of surface terms, built from critical syzygies as[5]

$$\mathrm{CSyzSurface}(\Gamma, \vec{\nu}) = \{S_\Gamma(\vec{a}, \vec{\nu}) : \vec{a} \in \pi(\mathrm{CSyz}_\Gamma)\}. \tag{3.37}$$

Interestingly, this construction gives a new perspective on the syzygy equation, eq. (2.10). In the case where $\mu = 1$ and $U^\Gamma_{\mathrm{crit}[\log(B)]}$ is isolated then it can be seen as a recipe to lift elements of the Lee-Pomeransky ideal $J^\Gamma_{\mathrm{LP}}$ to surface terms.

The importance of the set of critical surface terms arises as we have constructed it to be a set of surface terms that is sufficient in the large-$\epsilon$, maximal-cut limit and therefore can be used as a tool to fill the space of surface terms and perform a reduction to master integrals. To see this explicitly, consider that $S_\Gamma(\vec{a})$ reduces to $\mathfrak{c}(\vec{a})$ in the large-$\epsilon$ limit. Looking to eq. (3.37), we therefore see that the set of critical surface terms becomes the set of maximal-cut $a_0$ terms of $\mathrm{CSyz}(\Gamma)$ in this limit. As this set of terms is $A_0^\Gamma/J^\Gamma_{\mathrm{cut}}$ by eq. (3.35), we therefore see that $\mathrm{CSyzSurface}(\Gamma, \vec{\nu})$ is isomorphic to $A_0^\Gamma/J^\Gamma_{\mathrm{cut}}$. If we now consider eq. (3.30), we see that, given $\mu = 1$ and $U^\Gamma_{\mathrm{crit}[\log(B)]}$ a finite collection of points, we have that

$$\mathrm{CSyzSurface}(\Gamma, \vec{\nu}) \simeq \mathrm{Surface}(\Gamma, \vec{1}) / (\mathrm{Surface}(\Gamma, \vec{1}) \cap J^\Gamma_{\mathrm{cut}}), \tag{3.38}$$

where we have made use of the fact that, for two vector spaces $V$ and $W$ that are subspaces of some larger space, $(V + W)/W \simeq V/(V \cap W)$. Restricting now to the $\vec{\nu} = \vec{1}$ case, we see that by eq. (3.37) and eq. (3.38) we have that $\mathrm{CSyzSurface}(\Gamma, \vec{1})$ is a subspace of $\mathrm{Surface}(\Gamma, \vec{1})$ that is isomorphic to $\mathrm{Surface}(\Gamma, \vec{1})$, modulo surface terms that vanish on the maximal cut. Naturally, these surface terms that vanish on the cut can be captured by repeating the procedure for pinch topologies. Therefore, by iteratively constructing the set of all $\mathrm{CSyzSurface}(\Gamma_k, \vec{1})$ for all $\Gamma_k \subseteq \Gamma$, one can construct the full set of surface terms.

[5] We note that the space $\mathrm{CSyzSurface}(\Gamma, \vec{\nu})$ depends on the choice of $\pi$. However, under the conditions of $U^\Gamma_{\mathrm{crit}[\log(B)]}$ being points and $\mu = 1$, the space depends only on $\pi$ through surface terms which vanish on the maximal cut. We therefore choose to suppress $\pi$ in the notation.

JHEP02(2026)004

### 3.5 Multiplicity structure

To close out our discussion of syzygies and critical surface terms, we return to interpreting the case where the saturation index $\mu \neq 1$, that is, where the multiplicity of the $B = 0$ component of the syzygy ideal $J^{\Gamma}_{\text{syz}}$ is not 1. In the analysis of section 3.3, this stopped us from being able to conclude that critical syzygies provide all relations among Feynman integrals. In the following, we argue that by expanding our construction, we can find further relations. To this end, we consider an alternative construction of the syzygy formalism. Rather than starting directly from a judiciously chosen syzygy equation such as eq. (2.10), we make contact with the definition of surface terms in eq. (2.8). The driving observation is that when we construct total derivatives from the syzygy of eq. (2.10) we implicitly restrict the structure of our total derivatives. Specifically, we assume that the exponent of the Baikov polynomial inside the total derivative is the same as that of the integrals that are targeted for reduction. While the constraint is natural, releasing this constraint can allow for more integral relations, as we will see.

We begin by considering numerator polynomials $\mathcal{N}$ that arise from total derivatives as

$$\frac{\mathcal{N} B^{\gamma}}{\prod_{e\in\text{props}(\Gamma)} z_e} = \sum_{i\in\text{ISPs}(\Gamma)} \partial_i \left[\frac{B^{\gamma-\Delta} a_i}{\prod_{e\in\text{props}(\Gamma)} z_e}\right] + \sum_{e'\in\text{props}(\Gamma)} \partial_{e'} \left[\frac{B^{\gamma-\Delta} z_{e'} \overline{a}_{e'}}{\prod_{e\in\text{props}(\Gamma)} z_e}\right]. \tag{3.39}$$

Here, we introduce directly the propagator non-doubling constraint by including a factor of $z_{e'}$ in the numerator of the second term. Note that here we have made use of unit propagator powers for ease of analysis. Importantly, in eq. (3.39), we have introduced a (non-negative) integer $\Delta$. If $\Delta = 0$, then this corresponds to the syzygy analysis in section 2, while $\Delta > 0$ is more general. From the perspective of the Laporta algorithm, eq. (3.39) makes use of "seed" integrals (those of which we take the derivative) with a power of the Baikov polynomial that is lower than that of the target integrals. Recalling that $\gamma = (D - E - l - 1)/2$, one effectively considers seed integrals defined in $D - 2\Delta$ dimensions, rather than $D$ dimensions. Let us expand out the argument of the total derivative and remove the common factor of $\frac{B^{\gamma-\Delta-1}}{\prod_{e\in\text{props}(\Gamma)} z_e}$, leading to

$$\mathcal{N} B^{\Delta+1} = (\gamma - \Delta) \left[\sum_{i\in\text{ISPs}(\Gamma)} a_i \partial_i B + \sum_{e\in\text{props}(\Gamma)} \overline{a}_e z_e \partial_e B\right] + B \left[\sum_{i\in\text{ISPs}(\Gamma)} \partial_i a_i + \sum_{e\in\text{props}(\Gamma)} z_e \partial_e \overline{a}_e\right]. \tag{3.40}$$

Next, we must impose that the right-hand side of eq. (3.40) is proportional to $B^{\Delta+1}$. We see that this takes on a different character, depending on the value of $\Delta$. If $\Delta = 0$, then the second term on the right-hand side of eq. (3.40) is already proportional to $B$ and so one only has the constraint that the first term is proportional to the Baikov polynomial. This leads to the style of syzygy in eq. (2.10).

Let us consider making the right-hand side of eq. (3.40) proportional to $B^{\Delta+1}$ for general $\Delta$. We see that the second term can no longer be ignored and that the proportionality constraints now involve not only syzygy-like terms, but also terms involving derivatives of the $a_i$ and $\overline{a}_e$. Such constraints provide an interesting challenge, but we leave direct understanding of the mathematical structure of their solution to future work. To make progress, we, therefore, consider restricting to $\gamma$ independent solutions for the $a_i$ and $\bar{a}_e$. While not the general class of solutions to eq. (3.40), this strategy is automatic in the $\Delta = 0$ case and we consider it more generally due to its success there. This allows us to break down the proportionality constraint into two separate constraints

$$A^{[\Delta,0]}B^{\Delta} = \sum_{i\in\mathrm{ISPs}(\Gamma)} \partial_i a_i + \sum_{e\in\mathrm{props}(\Gamma)} z_e \partial_e \bar{a}_e, \tag{3.41}$$

$$A^{[\Delta,1]}B^{\Delta+1} = \sum_{i\in\mathrm{ISPs}(\Gamma)} a_i \partial_i B + \sum_{e\in\mathrm{props}(\Gamma)} \bar{a}_e z_e \partial_e B, \tag{3.42}$$

where we impose that the $A^{[\Delta,i]}$ are polynomial. Note that, for $\Delta = 0$, the constraint that $A^{[\Delta,0]}$ is polynomial, is solved for any values of $a_i$ or $\bar{a}_e$. However, for higher values of $\Delta$, this constraint is non-trivial.

Let us consider the two proportionality constraints in turn. First, we see that eq. (3.41) depends on the derivatives of the unknown polynomials. If we take it both on the maximal cut and the zero locus of the Baikov polynomial, that is, we set $B = z_e = 0$, we see that eq. (3.41) can be read as a statement that vector field of the $a_i$ is divergenceless. The second constraint, eq. (3.42) depends linearly on the unknown polynomials, and so is again a syzygy constraint. We can make an analogous analysis to section 3.2 by decomposing $A^{[\Delta,1]}$ into an on-shell and off-shell part as

$$A^{[\Delta,1]} = a_0^{[\Delta,1]} + \sum_{e\in\mathrm{props}(\Gamma)} \tilde{a}_0^{[\Delta,1]} z_e. \tag{3.43}$$

The requirement that the $a_0^{[\Delta,1]}$ is polynomial therefore implies the constraint that $a_0^{[\Delta,1]}$ belongs to the ideal

$$A_0^{\Gamma,\Delta} = J^{\Gamma}_{\mathrm{syz},\Delta} : \langle B^{1+\Delta}\rangle, \tag{3.44}$$

where

$$J^{\Gamma}_{\mathrm{syz},\Delta} = \Big\langle \partial_i B \ : \ i \in \mathrm{ISPs}(\Gamma)\rangle + \langle z_e\partial_e B,\, z_e B^{1+\Delta} \ : \ e \in \mathrm{props}(\Gamma)\Big\rangle. \tag{3.45}$$

Note that this constraint is a generalization of the discussion of section 3.2, as $A_0^{\Gamma,0} = A_0^{\Gamma}$. Moreover, note that if we use eq. (3.40) to build the associated surface term $\mathcal{N}$, then the large-$\epsilon$, maximal cut limit is $a_0^{[\Delta,1]}$. Therefore, we again see that, in the large-$\epsilon$, maximal cut limit, a surface term belongs to an ideal.

We can develop further insight into the meaning of $\Delta$ by observing that we can rewrite $A_0^{\Gamma,\Delta}$ as[6]

$$A_0^{\Gamma,\Delta} = [\langle \partial_i B \ : \ i \in \mathrm{ISPs}(\Gamma)\rangle + \langle z_e\partial_e B \ : \ e \in \mathrm{props}(\Gamma)\rangle] : \langle B^{1+\Delta}\rangle \ + \ J^{\Gamma}_{\mathrm{cut}}. \tag{3.46}$$

This representation of $A_0^{\Gamma,\Delta}$ tells us that $\Delta$ controls the power of the Baikov polynomial in the quotient of the inner ideal of eq. (3.46). We therefore see that there exists a finite $\overline{\Delta}$ for which $A_0^{\Gamma,\Delta}$ stabilizes, which can be recognized as the saturation index of the quotient in eq. (3.46).

[6]This follows by the more general identity that for two $R$-ideals $J$, $K$ and $x$, an element of $R$, one has that $(J + Kx^N) : \langle x^N\rangle = J : x^N + K$, which can be easily proven by simple two-sided inclusion arguments.

JHEP02(2026)004

Moreover, we see that $A_0^{\Gamma,\Delta}$ may be larger than $A_0^{\Gamma}$ as quotienting by higher powers may lead to new elements of the ideal. As we have recognized $A_0^{\Gamma,\Delta}$ as the set of surface terms in the large $\epsilon$, on-shell limit, we can expect this construction to lead to more surface terms in cases where $\overline{\Delta} > 0$. We will see in section 5.2 that there do exist physical examples where $\overline{\Delta} \neq 0$, and, correspondingly, that this construction leads to more surface terms.

## 4 One-loop critical syzygies

Having introduced the theory of critical syzygies in the previous section, here we begin their practical study by considering them at one loop. At this loop order, it is well understood that there is at most one master integral associated to each topology. In this section, we will discuss how this statement arises by using critical syzygies to explicitly construct surface terms. An important practical aspect that we study is that of power-counting constraints. It is well known that the Feynman rules of gauge theory lead to an upper bound on the total polynomial degree of the numerators that one must consider in an amplitude calculation. Specifically, letting $|\Gamma|$ be the number of edges in $\Gamma$, the numerator associated to $\Gamma$ is a polynomial in

$$R^{(\Gamma,1)} = \{\, p \in R \ : \ \deg(p) \leq |\Gamma| \,\}\,, \tag{4.1}$$

where $\deg(p)$ is the total polynomial degree of $p$ in Baikov variables and the index 1 in $R^{(\Gamma,1)}$ denotes that it is the one-loop power-counting space. Concretely, our aim is to construct a basis of the space of critical surface terms for Feynman integrals with unit propagator powers that are compatible with power counting, i.e. a basis of $\mathrm{CSyzSurface}(\Gamma, \vec{1}) \cap R^{(\Gamma,1)}$.

To begin our analysis, let us consider the discussion at the end of section 3.2, which tells us that non-trivial calculation is only required if the zero-locus of the $\Gamma$-cut of the Baikov polynomial is a smooth surface. Explicitly we must check if $U^{\Gamma}_{\mathrm{sing}}$ is empty, that is, if there are any solutions to eq. (3.5) for $\Gamma_i = \Gamma$. To understand this, an important observation is to recall that, at one loop, the Baikov polynomial is at most quadratic in each Baikov variable. Without loss of generality, we will order the Baikov parameters such that $z_0, \ldots, z_{I-1}$ label the $I$ irreducible scalar products and $z_I, \ldots, z_{N-1}$ label the propagators. It is then easy to write the one-loop Baikov polynomial as

$$B = \frac{1}{2}(\vec{z}_i, \vec{z}_e, 1) \begin{pmatrix} \mathscr{H}_\Gamma & X & \vec{\mathscr{B}}_i \\ X^T & O & \vec{\mathscr{B}}_e \\ \vec{\mathscr{B}}_i^{\,T} & \vec{\mathscr{B}}_e^{\,T} & \mathscr{B}_0 \end{pmatrix} \begin{pmatrix} \vec{z}_i \\ \vec{z}_e \\ 1 \end{pmatrix}, \tag{4.2}$$

where we gather the ISPs and propagators into $\vec{z}_i$ and $\vec{z}_e$ respectively and the $\mathscr{H}_\Gamma$ and $O$ are symmetric matrices of side-length $I$ and $N-I$ respectively. Notice that the matrix $\mathscr{H}_\Gamma$ has been defined so that it is the Hessian of the cut Baikov polynomial, i.e.

$$(\mathscr{H}_\Gamma)_{ij} = \partial_i \partial_j B|_{\mathrm{cut}_\Gamma}. \tag{4.3}$$

The representation of eq. (4.2) allows us to easily compute the partial derivatives of the Baikov polynomial with respect to an ISP as

$$\vec{\partial}_i B = \mathscr{H}_\Gamma \vec{z}_i + X \vec{z}_e + \vec{\mathscr{B}}_i. \tag{4.4}$$

JHEP02(2026)004

It is therefore clear that the critical locus of the Γ-cut Baikov polynomial at one loop is given by an intersection of hyperplanes. The dimensionality of this configuration is controlled by the rank of the rectangular matrix $(\mathscr{H}_\Gamma\ \vec{\mathscr{B}}_i)$. For the moment, we will proceed with the assumption that this rank is maximal, and so the critical locus is given by a single point. In this situation we can solve for the ISPs as a function of the Baikov derivatives, which allows us to rewrite the Baikov polynomial on the maximal cut as

$$B|_{z_e=0\,:\,e\in\text{props}(\Gamma)} = \frac{1}{2}(\vec{\partial}_i B, 1)\begin{pmatrix} \mathscr{H}_\Gamma^{-1} & 0 \\ 0 & \mathscr{B}_0 - \vec{\mathscr{B}}_i^{\,T}\mathscr{H}_\Gamma^{-1}\vec{\mathscr{B}}_i \end{pmatrix}\begin{pmatrix} \vec{\partial}_i B \\ 1 \end{pmatrix} \tag{4.5}$$

Taking eq. (4.5) on $B = \partial_i B = 0$, we see that the smoothness condition is translated into the algebraic constraint on the external kinematics that the constant term in eq. (4.5) is non-zero.

### 4.1 Regular cases

Let us begin with a class of critical syzygies that clearly arise without involved calculation. At one loop, they turn out to generate the full collection of surface terms in the case where the number of master integrals associated to Γ is 1, and are almost sufficient in the case where the number of master integrals is 0. They (non-manifestly) contain the set of surface terms in the OPP basis [55] as well as those that allow for reduction of $\epsilon$-dimensional numerators (see e.g. [29]). For each ISP $z_i$, let us consider a syzygy where

$$a_0 = \partial_i B, \quad a_i = -B, \quad a_{j\neq i} = 0, \quad \text{and} \quad \tilde{a}_e = \overline{a}_e = 0. \tag{4.6}$$

This clearly solves eq. (2.10) as all we have done is to take anti-symmetric combinations of the generators. Indeed, they are a subset of "principal syzygy" solutions to eq. (2.10). For this reason, we will denote each such principal critical syzygy as $\vec{a}_i^p$. As the solution set of eq. (2.10) has the structure of a module, we can multiply any solution by a polynomial and still get a solution. We therefore consider the syzygy $\lambda\vec{a}_i^p$ which gives rise to the surface term

$$S_\Gamma(\lambda\vec{a}_i^p) = \lambda\alpha_i + \frac{B\partial_i\lambda}{\gamma+1}, \tag{4.7}$$

where we implicitly use that all of the $\nu_i = 1$, suppressing the notation and define

$$\alpha_i = \partial_i B. \tag{4.8}$$

Due to the fact that, at one loop, the Baikov polynomial is quadratic in all variables, the $\alpha_i$ are degree 1 in Baikov variables. Moreover, for generic kinematics they are linearly independent. Therefore, they form a natural set of variables on the cut associated to Γ, and we will phrase our surface term construction in terms of them.

Let us now consider using the principal critical syzygies to build surface terms. The task is to choose an appropriate set of $\lambda$ such that we have a basis of the full space of associated surface terms on the cut associated to Γ, while remaining in the one-loop power counting space, $R^{(\Gamma,1)}$. To this end, let us first observe that, in the large-$\epsilon$ limit

$$\lim_{\epsilon\to\infty} S_\Gamma(\lambda\vec{a}_i^p) \in J^\Gamma_{\text{crit}(B)}. \tag{4.9}$$

JHEP02(2026)004

We can therefore regard the full $S_\Gamma(\lambda\vec{a}_i^p)$ as a prescription to lift an element of $J^\Gamma_{\text{crit}(B)}$ to a surface term. It is easy to see that, given that the $\alpha_i$ are linear in the $z_i$, $J^\Gamma_{\text{crit}(B)}/J^\Gamma_{\text{cut}}$ is just the space of all monomials in the $\alpha_i$ with degree at least 1. By this argumentation, through an appropriate choice of $\lambda$ and $i$ we can generate a surface term associated to every such monomial. These surface terms are clearly linearly independent, as their large-$\epsilon$ limit is a linearly independent set of monomials in $\alpha_i$. Moreover, as

$$\deg\left(S_\Gamma(\lambda\vec{a}_i^p)\right) = \deg\left(\lim_{\epsilon\to\infty} S_\Gamma(\lambda\vec{a}_i^p)\right) = \deg(\lambda) + 1, \tag{4.10}$$

we see that, first, the lifting procedure from a monomial to a surface term does not change the power counting away from that of the large $\epsilon$ limit and, second, by consideration of eq. (4.1), we have a degree bound on the monomial $\lambda$ which is easy to satisfy.

To consider this procedure more concretely, let us construct a series of surface terms for a box diagram $\Gamma$ that is a subtopology of a top-level pentagon. To this end, we begin by working in the Baikov parameterization of the pentagon, where $\gamma = -1-\epsilon$. Power counting limits us to at most degree 4 numerator polynomials for the box. The associated set of surface terms is then

$$\left\{\alpha_0^{n+1} - \frac{n}{\epsilon}B\alpha_0^{n-1}(\partial_0)^2 B \quad : \quad n \in \{0,1,2,3\}\right\}, \tag{4.11}$$

where we denote the ISP of the box as $\alpha_0$. It is clear that all of these functions are linearly independent and that they are linearly independent of the scalar integral, which we can take as our master, as expected.

## 4.2 Singular cases

From the discussion at the top of the section, there are naturally two situations where the non-singularity of the Baikov polynomial on the maximal cut comes into question. The first is when the Hessian of the cut Baikov polynomial is not invertible. The second is when the constant term in eq. (4.5) vanishes, and hence the Baikov polynomial gives a singular variety. Let us consider these two cases in turn.

Firstly, we consider the Hessian of the cut Baikov. In order to do this, we write the Baikov polynomial in a special form, making use of the well-known "Cayley-Menger" trick.[7] To employ the Cayley-Menger trick, let us begin by defining the momenta $q_k$ and masses $m_k$ as those associated to the Baikov variable $z_k$. That is,

$$z_k = (\ell - q_k)^2 - m_k^2. \tag{4.12}$$

This allows us to write the Baikov polynomial as

$$B = \frac{(-1)^{N+1}}{2^N}\det\begin{pmatrix} 0 & z_0 & \dots & z_{N-1} & 1 \\ z_0 & C_{00} & \dots & C_{(N-1)0} & 1 \\ \vdots & \vdots & \dots & \vdots & \vdots \\ z_{N-1} & C_{(N-1)0} & \dots & C_{(N-1)(N-1)} & 1 \\ 1 & 1 & \dots & 1 & 0 \end{pmatrix}, \tag{4.13}$$

[7]This trick is intimately related to the so-called "Embedding-space formalism" [29, 34].

JHEP02(2026)004

where $C_{kl} = (q_k - q_l)^2 - m_k^2 - m_l^2$ is the so-called "Cayley matrix". The benefit of this notation is that it allows us to write the entries of the matrices in eq. (4.2) as minors of the matrix

$$\mathscr{C} = \begin{pmatrix} C & \vec{1} \\ \vec{1}^T & 0 \end{pmatrix}, \tag{4.14}$$

where the $\vec{1}$ represents a column containing just 1s. Note that, again by the Cayley-Menger trick, we have that

$$G(q_1, \dots q_{N-1}) = \frac{(-1)^N}{2^{N-1}} \det(\mathscr{C}), \tag{4.15}$$

i.e. it is the Gram determinant associated to the Baikov parameterization. We will denote the minor of $\mathscr{C}$ where row $i$ and column $j$ have been removed as $\mathscr{C}[\hat{\imath}, \hat{\jmath}]$. With this notation, differentiation of eq. (4.13) gives

$$(\mathscr{H}_\Gamma)_{ij} = \frac{(-1)^{i+j+N}}{2^{N-1}} \mathscr{C}[\hat{\imath}, \hat{\jmath}], \qquad i, j \in [0, \dots, I-1]. \tag{4.16}$$

To understand if $\mathscr{H}_\Gamma$ is invertible, we compute its determinant. By Jacobi's theorem on complementary minors this is given by

$$\det(\mathscr{H}_\Gamma) = \left[\frac{(-1)^N}{2^{N-1}}\right]^I \det(\mathscr{C})^{I-1} G_\Gamma, \tag{4.17}$$

where

$$G_\Gamma = \det\left(\mathscr{C}_{ef}, \qquad e, f \in [I, \dots, N-1, N]\right) \tag{4.18}$$

is the determinant of the minor of $\mathscr{C}$ corresponding to the propagators of $\Gamma$. By the Cayley-Menger trick, we have that this determinant is a constant multiple of the Gram determinant that we associate to $\Gamma$.[8] The case where $\det(\mathscr{H}_\Gamma)$ vanishes because $\det(\mathscr{C})$ vanishes is not of interest as, looking to eq. (2.1), we see that it corresponds to a region of phase-space where the Baikov parameterization itself is degenerate. We therefore conclude that the critical locus of the Baikov polynomial at one loop can only be non-isolated if the Gram determinant of the external momenta of $\Gamma$ is zero. For fixed-angle scattering at one loop, this can occur in only one case where the associated integrals are not scaleless: the "external leg correction"-bubble that we depict in figure 1. In practice, it turns out that all maximal minors of the rectangular matrix $(\mathscr{H}_\Gamma \; \vec{B}_i)$ vanish and hence the critical locus of this topology is not a finite set of points. In such cases, critical syzygies are insufficient for a complete reduction to master integrals. We therefore leave further study to future work.

If the Hessian of the Baikov matrix is of full rank on the maximal cut, then the critical locus is isolated. As stated earlier, it remains to check the non-singularity condition. To this end, let us rewrite the constant term of eq. (4.5) by recognizing it as a Schur complement as

$$\det\begin{pmatrix} \mathscr{H}_\Gamma & \vec{\mathscr{B}}_i \\ \vec{\mathscr{B}}_i^T & \mathscr{B}_0 \end{pmatrix} = \left(\mathscr{B}_0 - \vec{\mathscr{B}}_i{}^T \mathscr{H}_\Gamma^{-1} \vec{\mathscr{B}}_i\right) \det(\mathscr{H}_\Gamma). \tag{4.19}$$

[8] We define $G_\Gamma$ of a tadpole graph, that involves no momenta, to be 1.

JHEP02(2026)004

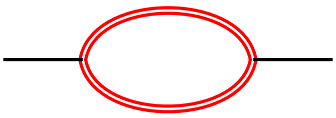

**Figure 1.** The single one-loop graph in fixed-angle scattering for which the critical locus is not isolated. The red lines represent a field of mass $m$ (e.g. a top). The black lines represent massless particles, that is $p^2 = 0$. The associated Feynman integral is only not scaleless for $m \neq 0$.

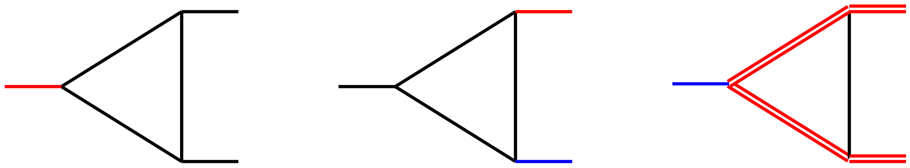

**Figure 2.** Representative examples of one-loop diagrams which have vanishing Cayley determinant. Black lines represent massless particles. Doubled red lines represent massive particles. Blue or single-red lines represent off-shell particles. For these examples, the set of critical syzygies is larger than the set generated by principal syzygies and further computation is required.

It therefore remains to compute the determinant on the left-hand-side. This is easily achieved using the same logic as the computation of $\det(\mathscr{H}_\Gamma)$, only noting that we now include the last row and column of $\mathscr{C}$, hence we have that

$$\det\begin{pmatrix} \mathscr{H}_\Gamma & \vec{\mathscr{B}}_i \\ \vec{\mathscr{B}}_i^T & \mathscr{B}_0 \end{pmatrix} = \left[\frac{(-1)^N}{2^{N-1}}\right]^{I+1} \det(\mathscr{C})^I C_\Gamma, \tag{4.20}$$

where

$$C_\Gamma = \det(C_{ef}, \qquad e,f \in [I,\dots,N-1]), \tag{4.21}$$

is the minor of the Cayley-matrix $C$, associated to the propagators that are cut. Altogether, we find that we can write

$$\mathscr{B}_0 - \vec{\mathscr{B}}_i{}^T \mathscr{H}_\Gamma^{-1} \vec{\mathscr{B}}_i = \frac{(-1)^N}{2^{N-1}} G(q_1,\dots,q_{N-1}) \frac{C_\Gamma}{G_\Gamma}. \tag{4.22}$$

We therefore see that the zero locus of the $\Gamma$-cut Baikov polynomial is a singular variety either if $G(q_1,\dots,q_{N-1}) = 0$ or if $C_\Gamma = 0$. As earlier, we discard the zero-Gram case and consider the more interesting case, $C_\Gamma = 0$. It is well-known that this corresponds to the occurrence of first-type Landau singularities (see, e.g. [56] for recent discussion). Examples of diagrams that exhibit this phenomenon are infra-red divergent triangle graphs, such as those that we exemplify in figure 2. Here, the set of critical syzygies becomes larger than the set generated by principal syzygies. Specifically, as the critical locus is already a point, we expect that there is exactly one interesting non-principal syzygy. Concretely, the ideal generated by the partial derivatives does not contain the constant polynomial, whereas $A_0^\Gamma$ does, hence there must be a syzygy where $a_0$ is a constant. This is most easily displayed in the Baikov representation associated to the cut, that is, the one where there are no ISPs. Considering the case of the one-mass triangle in figure 2, we explicitly find the syzygy

$$0 = a_0 B + \bar{a}_1 z_1 \partial_1 B + \bar{a}_2 z_2 \partial_2 B + \bar{a}_3 z_3 \partial_3 B + \tilde{a}_2 z_2 B, \tag{4.23}$$

JHEP02(2026)004

where the components of the syzygy are given by

$$\begin{aligned} a_0 &= -2s^2, \\ \bar{a}_1 &= s^2 - s[z_1 - z_3] + 4z_2[2s - 2z_3 + z_2 - z_1], \\ \bar{a}_2 &= 2s^2 + 6sz_2 + 4z_2^2 + s(z_1 + z_3) - 4[z_1z_2 + z_3z_2 + z_1z_3], \\ \bar{a}_3 &= s^2 - s[z_3 - z_1] + 4z_2[2s - 2z_1 + z_2 - z_3], \\ \tilde{a}_2 &= -8(s + z_2 - z_1 - z_3). \end{aligned} \tag{4.24}$$

It would be interesting to understand if the full syzygy could be determined from geometrical arguments, but we leave this to future work. This syzygy then gives rise to a surface term,

$$S_\Gamma(\vec{a}) = -2s^2 - 8z_2(s + z_2 - [z_1 + z_3])\left(1 - \frac{1}{\gamma}\right) + \frac{s}{\gamma}(z_1 + z_3 + 2z_2), \tag{4.25}$$

which manifestly becomes constant on the triangle cut $z_1 = z_2 = z_3 = 0$, and hence provides a reduction relation for the scalar triangle.

## 5 Critical surface terms at two loops

The construction of critical surface terms at two loops is a more complicated affair than at one loop. At two loops, the structure of the Baikov polynomial is more involved than a quadric, and so we are more constrained in our ability to analytically construct critical surface terms. In this section, we study the question: can we computationally use critical surface terms to perform reduction to master integrals? While surface terms from standard syzygies have been found to be sufficient in many calculations, as critical surface terms are effectively a subset, this question should be studied. As at one loop, in practical applications to two-loop integral reduction, surface terms are constructed satisfying power-counting constraints. For concreteness, we again adopt gluonic power counting, defining the two-loop power-counting space of numerator polynomials associated to a diagram $\Gamma$ as

$$R^{(\Gamma,2)} = \{a \in R \quad : \quad \deg_{\ell_1}(a) \le |\Gamma_1|,\ \deg_{\ell_2}(a) \le |\Gamma_2|,\ \deg_{\ell_1}(a) + \deg_{\ell_2}(a) < |\Gamma|\}\,, \tag{5.1}$$

where $|\Gamma_i|$ is the number of edges in $\Gamma$ that depends on loop momentum $i$, $|\Gamma|$ is the total number of edges in $\Gamma$ and $\deg_{\ell_i}(\mathrm{a})$ is the polynomial degree of $a$ in loop momentum $i$. In order to reduce the integrals arising in a QCD amplitude that are associated to the set of propagators $\Gamma$ and exponents $\vec{\nu}$, an integral reduction program needs to explicitly construct elements of

$$\mathrm{Surface}^R(\Gamma, \vec{\nu}) = \mathrm{Surface}(\Gamma, \vec{\nu}) \cap R^{(\Gamma,2)}, \tag{5.2}$$

surface terms that live within power counting. The goal of this section is to study computational construction of surface terms from critical syzygies. Naturally, the isomorphism in eq. (3.38) implies that, if a diagram has isolated critical points and $\mu = 1$, critical surface terms are in principle sufficient to reduce all tensor integrals associated to this diagram. However, it is *a priori* unclear if it is possible to construct a finite-dimensional restriction of $\mathrm{CSyzSurface}(\Gamma, \vec{\nu})$ that lives within power counting in a finite number of steps. That

JHEP02(2026)004

is, can we computationally construct a set of critical syzygies that give rise to a subspace $\mathrm{CSyzSurface}^R(\Gamma, \vec{\nu}) \subset \mathrm{Surface}^R(\Gamma, \vec{\nu})$ such that

$$\mathrm{CSyzSurface}^R(\Gamma, \vec{\nu}) \simeq \mathrm{Surface}^R(\Gamma, \vec{\nu})/[\mathrm{Surface}^R(\Gamma, \vec{\nu}) \cap J^\Gamma_{\mathrm{cut}}]? \tag{5.3}$$

In this section, we study this question in non-trivial examples and thereby provide evidence that critical surface terms are a useful tool for the reduction of tensor integrals in gauge theory. Unlike in the one-loop case, generic analytic construction of surface terms is a non-trivial problem. Indeed, at two loops, one experimentally finds that $B = 0$ on the maximal cut is a singular variety. That is, $U^\Gamma_{\mathrm{sing}}$ is never empty, and, therefore, principal syzygies are insufficient. For this reason, to study the question posed in eq. (5.3), we set up an algorithm to determine critical syzygies within power counting. We then consider a cutting-edge example: the leading-color contributions to the two-loop $t\bar{t}H$ amplitude. We demonstrate that, for the light-quark contributions (whose master integrals were recently computed [57]) the critical locus is always a finite set of points and that the $\mu = 1$ hypothesis holds in all but one case. In this way we are able to provide a positive answer to the question raised in eq. (5.3) in a physically interesting example.

### 5.1 Computational construction of critical surface terms

Let us now turn to the question of constructing a basis of critical surface terms that are compatible with the power-counting constraints. The practical strategy that we will employ is to take the definition of $\mathrm{CSyzSurface}(\Gamma, \vec{\nu})$ in eq. (3.37) as a prescription to construct critical surface terms. Specifically, we will explicitly construct a finite subspace $\mathrm{CSyz}^R(\Gamma) \subset \mathrm{CSyz}(\Gamma)$, and define

$$\mathrm{CSyzSurface}^R(\Gamma, \vec{\nu}) = \{S_\Gamma(\vec{a}, \vec{\nu}) : \vec{a} \in \pi(\mathrm{CSyz}^R(\Gamma))\}. \tag{5.4}$$

Furthermore, we will constrain the basis of $\pi(\mathrm{CSyz}^R(\Gamma))$ such that all surface terms are within power counting. An important observation is that this construction of $\mathrm{CSyzSurface}^R(\Gamma, \vec{\nu})$ develops a strong dependence on the choice of $\pi$. To see this, consider specifying $\pi$ by specifying a basis $\{[\vec{a}_1], \ldots\}$ of $\mathrm{CSyz}(\Gamma)$ and their associated lifts $\vec{a}_i \in \mathrm{Syz}(\Gamma)$. Note that, for any choice of lift $\vec{a}$ of some $[\vec{a}]$, the large-$\epsilon$, on-shell limit of $S_\Gamma(\vec{a}, \vec{\nu})$ is independent of the specific choice. However, the polynomial degree of $S_\Gamma(\vec{a}, \vec{\nu})$ does depend on the specific choice of the lift, through the sub-leading terms in the large-$\epsilon$, on-shell limit. We can therefore see that to computationally construct $\mathrm{Surface}^R(\Gamma, \vec{\nu})$ via critical syzygies will require carefully choosing the lifts.

A conceptually simple way to construct $\mathrm{CSyz}^R(\Gamma)$ is to write an ansatz for some $\vec{a}$ that is a syzygy whose associated surface term lives within power counting. One then uses linear algebra to find a basis of such terms which are linearly independent in the large-$\epsilon$, on-shell limit. Nevertheless, due to the high polynomial degrees involved, this is a computationally demanding approach. Instead, we construct an approach to generate critical surface terms which exploits the module properties of $\mathrm{CSyz}(\Gamma)$, thereby keeping the computation tractable. Specifically, we break the problem down into two steps. We first construct a generating set of $\mathrm{CSyz}(\Gamma)$ using linear algebra methods given by low-degree representatives in $\mathrm{Syz}(\Gamma)$. We then construct a basis of $\mathrm{CSyz}^R(\Gamma)$ by taking polynomial combinations of our generating set,

JHEP02(2026)004

constraining the combinations such the associated surface terms satisfy the power-counting constraints and are linearly independent in the large-$\epsilon$, on-shell limit. In the following, we elaborate on the details of each component of our approach, highlighting tricks for reducing the size of the involved linear systems. We provide a summary of the approach in section 5.1.3.

### 5.1.1 Constructing a generating set of critical syzygies

Let us consider the question of constructing a generating set of the module of critical syzygies, $\mathrm{CSyz}(\Gamma)$, defined in eq. (3.34). Our approach will be to use linear algebra to construct a generating set $\{[\vec{v}_1], \ldots\}$ of $\mathrm{CSyz}(\Gamma)$ by finding representatives $\vec{v}_i$ of these elements in $\mathrm{Syz}(\Gamma)$. Note that a generating set of a module is not unique and it is a non-trivial problem to construct a minimal generating set of a module. Instead, we will construct a generating set of $\mathrm{CSyz}(\Gamma)$ up to a input power-counting bound. This is naturally not a minimal construction and, therefore, the number of generators will depend on this bound. We will then check if this set generates $\mathrm{CSyz}(\Gamma)$ by exploiting the isomorphism of $\mathrm{CSyz}(\Gamma)$ to $A_0^\Gamma / J_{\mathrm{cut}}^\Gamma$.

We begin by expressing all unknowns of the syzygy equation, eq. (2.10), as polynomials in Baikov variables with coefficients that are rational functions of kinematics. Writing all unknowns in eq. (2.10) as some $a_j$ for simplicity, we parameterize them as

$$a_j = \sum_{k:|\vec{\alpha}_k| \leq N_j} c_{jk}(\vec{s}) Z^{\vec{\alpha}_k}, \tag{5.5}$$

where we write a monomial in Baikov variables as

$$Z^{\vec{\alpha}} := \prod_{m=1}^{N} z_m^{\alpha_m}, \quad \alpha_m \in \mathbb{Z}_{\geq 0}, \tag{5.6}$$

$|\vec{\alpha}| = \sum_m \alpha_m$ is the total degree and the coefficients $c_{jk}$ are unknown rational functions in external kinematics $\vec{s}$.[9] In eq. (5.5), we denote the total degree of $a_j$ in Baikov variables as $N_j \in \mathbb{Z}_{\geq 0}$. The set of $N_j$ are the input power-counting bounds to our procedure and control its computational cost. A useful computational observation is that there are a number of easily identifiable syzygies $\vec{a}$ which lead to $S_\Gamma(\vec{a}, \vec{\nu}) = 0$. Specifically, this is the set of syzygies, $\mathrm{ZSyz}(\Gamma)$ discussed in section 3.4. Such syzygies can be seen as an artefact of our decision to break up the coefficient of the Baikov polynomial in eq. (2.10) into an on-shell part, $a_0$ and off-shell parts, the $\tilde{a}_e$, which was useful for the analysis of section 3.2. Nevertheless, computationally, such syzygies are irrelevant and we remove them from the ansatz by letting $a_0$ depend only on ISPs and $\tilde{a}_e$ depend on ISPs and only on propagators $z_{e'}$ such that $e' \leq e$.

By inserting the ansatz eq. (5.5) into the syzygy equation eq. (2.10) and requiring that the coefficient of each monomial in Baikov variables should vanish, we convert a linear system with polynomial unknowns $a_k$ into a linear system of unknown rational functions $c_{jk}$. That is, gathering all unknowns $c_{jk}$ of eq. (5.5) into the object $C = \bigcup_{j,k} c_{jk}$, we reparametrize the syzygy equation eq. (2.10) as

$$\sum_j M_{ij} C_j = 0, \tag{5.7}$$

[9]While $\mathrm{CSyz}(\Gamma)$ is an $R$-module, and so elements of $\mathrm{CSyz}(\Gamma)$ can depend on $\epsilon$, the syzygy equation eq. (2.10) is independent of $\epsilon$ and so the generating set need not depend on $\epsilon$. Hence, choosing the $c_{jk}$ to be $\epsilon$ independent is no restriction.

JHEP02(2026)004

where $M$ is a matrix of rational functions of the kinematics and the index $i$ runs over all monomials in Baikov variables that arise when inserting the ansatz into eq. (2.10). By eq. (5.5), a basis of solutions to eq. (5.7) corresponds to a basis of the degree-bounded subspace of $\mathrm{Syz}(\Gamma)$ controlled by the $N_j$, which we denote as $\{\vec{v}_1, \ldots, \vec{v}_{C(\vec{N})}\}$. Naturally, each $\vec{v}_i$ is a representative of some $[\vec{v}_i]$ of $\mathrm{CSyz}(\Gamma)$. We note that, as representatives $\vec{v}$ that differ by an element with zero critical part are equivalent, the elements $\{[\vec{v}_1], \ldots, [\vec{v}_{C(\vec{N})}]\}$ are not necessarily linearly independent. Nevertheless, we choose to leave these redundancies in our system, as they will be tackled at the next stage.

The set $\{[\vec{v}_1], \ldots, [\vec{v}_{C(\vec{N})}]\}$ forms a natural guess for a generating set of the unbounded $\mathrm{CSyz}(\Gamma)$ as, for sufficiently high $\vec{N}$ it must generate the module, by the ascending chain condition. In order to check if we indeed do have a generating set of $\mathrm{CSyz}(\Gamma)$ we make use of the fact that it is isomorphic to $A_0^\Gamma / J_{\mathrm{cut}}^\Gamma$. Specifically, the set $\{[\vec{v}_1], \ldots, [\vec{v}_{C(\vec{N})}]\}$ generates $\mathrm{CSyz}(\Gamma)$ if and only if the critical parts of the $\vec{v}_i$ generate $A_0^\Gamma / J_{\mathrm{cut}}^\Gamma$. Equivalently,

$$\langle [\vec{v}_1], \ldots, [\vec{v}_{C(\vec{N})}] \rangle = \mathrm{CSyz}(\Gamma) \qquad \Longleftrightarrow \qquad \langle \mathfrak{c}(\vec{v}_1), \ldots, \mathfrak{c}(\vec{v}_{C(\vec{N})}) \rangle + J_{\mathrm{cut}}^\Gamma = A_0^\Gamma. \tag{5.8}$$

To check if we indeed have a generating set of $\mathrm{CSyz}(\Gamma)$, we can therefore check equality of the two ideals on the right-hand side of eq. (5.8). This can easily be performed by checking if their reduced Groebner bases are equal. For the ideal generated by critical parts of syzygies, this is easily performed in the computer algebra system `Singular` [42] as we have the generating set by construction. In order to compute a Groebner basis of $A_0^\Gamma$, we perform the ideal quotient of eq. (3.15) computationally, again using `Singular`. If the two Groebner bases are distinct then we do not have a generating set of $\mathrm{CSyz}(\Gamma)$, reflecting that the degree bounds $\vec{N}$ are too low. In this way, given $\vec{N}$ we construct a generating set of $\mathrm{CSyz}(\Gamma)$ or report that the degree bound is too low.

Having set up an algorithm for computing a generating set of $\mathrm{CSyz}(\Gamma)$, let us make some practical remarks. While it is trivial to obtain $M$ analytically, solving the system of equations analytically is highly non-trivial due to the large size of $M$. For this reason, we apply our approach numerically, at a given phase-space point. This approach allows for a number of optimizations that are frequently applied in finite-field-based approaches, which we record here for completeness. An important feature is that many of the $c_{jk}$ in eq. (5.5) are zero. This can be detected when solving eq. (5.5) on an initial, randomly-chosen, phase-space point $\vec{s}_0$ and imposing this constraint for later evaluations. That is, we impose

$$c_{jk}(\vec{s}_0) = 0 \qquad \Rightarrow \qquad c_{jk}(\vec{s}) = 0, \tag{5.9}$$

and, therefore, in practice we use the refined ansatz

$$a_j = \sum_{k : c_{jk}(\vec{s}_0) \neq 0} c_{jk}(\vec{s}) Z^{\vec{\alpha}_k}. \tag{5.10}$$

In eq. (5.7) this has the effect of removing the columns of $M$ that correspond to $C_j(\vec{s}_0) = 0$. Moreover, we decrease the number of unknowns even further by observing that the $c_{jk}$ (or equivalently the $C_j$) are $\mathbb{Q}$-linearly dependent. Analogous to the approach applied when reconstructing scattering amplitudes [58], with a small number of evaluations of the $C_j(\vec{s})$,

JHEP02(2026)004

we resolve the $\mathbb{Q}$-linear dependencies and write

$$C_j = \sum_k A_{jk} \tilde{C}_k, \tag{5.11}$$

where the entries of matrix $A$ are rational numbers and $\tilde{C}_k$ are a linearly independent subset of the $C_j$. Combining with eq. (5.7) leads to

$$\sum_l \tilde{M}_{jl} \tilde{C}_l = 0, \qquad \text{where} \qquad \tilde{M}_{jl} = \sum_{k: C_k(\vec{s}_0) \neq 0} M_{jk} A_{kl}. \tag{5.12}$$

That is, one only has to row reduce the matrix $\tilde{M}_{jl}$. These optimizations can result in linear systems that are hundreds of times smaller than eq. (5.7).

In summary, given a set of power-counting bounds $N_j$, we construct a generating set for $\mathrm{CSyz}(\Gamma)$ on a fixed phase-space point $\vec{s}$. The size of the generating set depends on $\vec{N}$ and is neither a Groebner basis, nor a minimal generating set. The generating set is specified analytically, though implicitly, through the linear equation system eq. (5.12), which can easily be solved numerically.

### 5.1.2 Critical surface terms within the power-counting window

Using the algorithm of the previous subsection, for each diagram and an appropriately chosen power-counting bound $\vec{N}$, we can determine a generating set of $\mathrm{CSyz}(\Gamma)$. It therefore remains to use these to construct a basis of $\mathrm{CSyzSurface}^R(\Gamma)$. The approach we will take to this problem is to build surface terms from polynomial combinations of our generators set of $\mathrm{CSyz}(\Gamma)$. That is, we look for syzygies $\vec{w}$ such that

$$\vec{w} = \sum_{j=1}^{C(\vec{N})} \lambda_j \vec{v}_j \qquad \text{and} \qquad S_\Gamma(\vec{w}, \vec{\nu}) \quad \in \quad R^{(\Gamma,2)}, \tag{5.13}$$

where the $\lambda_j$ are polynomials in $R$ and the $\vec{v}_j$ are representatives in $\mathrm{Syz}(\Gamma)$ of the $[\vec{v}_j]$ in $\mathrm{CSyz}(\Gamma)$. As the $\vec{v}_j$ are known, eq. (5.13) is a non-trivial constraint on the polynomials $\lambda_j$. In this section we discuss two methods for satisfying this constraint. In the first, we take monomial multiples of each generator $\vec{v}_j$ that satisfy eq. (5.13), finding that this is often sufficient to find a basis of surface terms. In the second, we write an ansatz for the $\lambda_j$ and then use linear algebra to find $\vec{w}$ such that all terms which violate power counting vanish, analogous to the approach used in ref. [47].

To begin, we parameterize our linear combination of the generators $\vec{w}$ as

$$\vec{w} = \sum_{j,k} \tilde{w}_{jk}(\vec{s}) Z^{\vec{\alpha}_j} \vec{v}_k. \tag{5.14}$$

Here, the sum over $j$ runs over a suitably large set of monomials in the Baikov variables. For practical purposes, we take this to be the set of monomials in ISPs that satisfy the power-counting constraints. Given the ansatz in eq. (5.14), we then consider constructing the surface term $S_\Gamma(\vec{w}, \vec{\nu})$. This can be written as a linear combination of monomials that live within power counting and those that do not. That is,

$$S_\Gamma\left(\vec{w}, \vec{\nu}\right) = \sum_{Z^{\vec{\alpha}_i} \in R^{(\Gamma,2)}} n_i(\vec{s}, \gamma, \tilde{w}) Z^{\vec{\alpha}_i} + \sum_{Z^{\vec{\alpha}_i} \notin R^{(\Gamma,2)}} \sum_{j,k} m_{ijk}(\vec{s}, \gamma) \tilde{w}_{jk} Z^{\vec{\alpha}_i}, \tag{5.15}$$

JHEP02(2026)004

where the $m_{ijk}$ and $n_i$ are rational functions of kinematics and $D$ and the $n_i$ are linear in the $\tilde{w}_{jk}$. In order to live within power counting, each term in eq. (5.15) that is not in $R^{(\Gamma,2)}$ must vanish. That is, we have

$$\sum_{j,k} m_{ijk}(\vec{s},\gamma)\tilde{w}_{jk} = 0, \tag{5.16}$$

which is a linear constraint on the ansatz parameters $\tilde{w}_{jk}$.

We consider finding a basis of critical syzygy solutions to eq. (5.16) in two separate ways. The first approach is combinatorical. Specifically, we enumerate all possible values of $j$ and $k$, setting $\tilde{w}_{jk} = 1$ and otherwise letting the entries of $\tilde{w}$ be zero. We then check if this $\tilde{w}$ satisfies eq. (5.16). Effectively, this amounts to taking $\vec{w} = Z^{\vec{\alpha}_j}\vec{v}_k$ and checking if it satisfies the conditions

$$\begin{aligned} w_0 + \sum_{e\in\text{props}(\Gamma)} \tilde{w}_e z_e &\in R^{(\Gamma,2)}, \\ \sum_{i\in\text{ISPs}(\Gamma)} \partial_i w_i + \sum_{e\in\text{props}(\Gamma)} z_e \partial_e \tilde{w}_e &\in R^{(\Gamma,2)}. \end{aligned} \tag{5.17}$$

In this way, we generate a collection of $\vec{w}$ which satisfy the power-counting bounds. Naturally, the associated set of $[\vec{w}]$ exhibit linear dependencies. We therefore select from our collection a subset with linearly independent $\mathfrak{c}(\vec{w})$, which is a simple linear algebra problem. Importantly, as $S_\Gamma(\vec{w},\vec{\nu}) \to \mathfrak{c}(\vec{w})$ in the large-$\epsilon$, on-shell limit, we see that this allows us to count how many independent surface terms associated to $\Gamma$ we have constructed. If this is equal to the number of surface terms on the maximal cut, we conclude that we have found a basis set of solutions to eq. (5.16) and therefore found a basis of $\text{Surface}^R(\Gamma,\vec{\nu})$ modulo pinches and so our set of critical syzygies is complete.

Naturally, this combinatorical approach of solving eq. (5.16) is not guaranteed to find all solutions, as it may be necessary to take non-trivial linear combinations of products of monomials and $\text{CSyz}(\Gamma)$ generators. Therefore, if the completeness test fails, we consider directly solving eq. (5.16) as a linear system. In order to easily identify a linearly independent set of critical surface terms from our solutions, we additionally require that the $\tilde{w}_{jk}$ are independent of $\epsilon$.[10] This again allows us to certify sufficiency of the basis of surface terms by looking only at the critical part of the involved syzygy. Writing $m_{ijk} = m^{(0)}_{ijk} + \frac{1}{\gamma}m^{(1)}_{ijk}$, we therefore require that the $\tilde{w}_{jk}$ satisfy

$$\sum_{j,k} m^{(0)}_{ijk}(\vec{s})\tilde{w}_{jk} = 0, \qquad \sum_{j,k} m^{(1)}_{ijk}(\vec{s})\tilde{w}_{jk} = 0, \tag{5.18}$$

where we recall the $m^{(X)}_{ijk}$ are matrices rational in the external kinematics. We solve eq. (5.18) via linear algebra methods which leads to a collection of syzygies $\vec{w}$ that live within power counting. We then select from this set of syzygies, a subset with linearly independent critical part, thereby finding a basis of $\text{CSyz}^R(\Gamma)$ and consequently, $\text{CSyzSurface}^R(\Gamma,\vec{\nu})$.

Analogous to the construction of the generating set of $\text{CSyz}(\Gamma)$ discussed in section 5.1.1, the linear equation system in eq. (5.18) can pose a non-trivial challenge to solve. We therefore

[10]Importantly, it can be shown that a basis of the $\epsilon$-independent solutions of eq. (5.16) is also a basis of the $\epsilon$-dependent solutions of eq. (5.16), so this practical trick causes no conceptual issues.

JHEP02(2026)004

close this subsection by discussing a number of computational optimizations. First, note that, in practice, the $m_{ijk}^{(X)}$ are constructed from a set of generators of CSyz(Γ) that are known on a numerical phase-space point $\vec{s}^{(0)}$. We therefore discuss solving eq. (5.18) at $\vec{s} = \vec{s}^{(0)}$. Next, we note that the matrices in eq. (5.18) are sparse and so we apply sparse linear algebra methods to solve them. The sparsity of the solution basis can therefore depend on the order in which the equations in eq. (5.18) are solved. Practically, we first solve one of the two $m^{(X)}$ systems and insert the solutions into the other in order to solve the full system, choosing the ordering based upon the sparsity of the final solution.

Having evaluated a basis of solutions to eq. (5.18) on a single phase-space point, we are able to use the structure of the resulting basis (and the sparsity properties that it inherited from our construction method) to ease its evaluation on further phase-space points. Letting the $\{\tilde{w}^{(1)}, \dots, \tilde{w}^{(M)}\}$ be a basis of solutions to eq. (5.18), we make two structural observations about the basis. First, it is clear that rotating the basis by any element of $\mathrm{GL}(M)$ will lead to a second basis $\{\tilde{w}'^{(1)}, \dots, \tilde{w}'^{(M)}\}$ that also satisfies eq. (5.18). One is only able to uniquely fix a basis after specifying this $\mathrm{GL}(M)$ freedom, which can be achieved by choosing an appropriate sub-matrix to be the identity matrix. In practice, an appropriate such choice is automatically performed when solving for the $w_{jk}^{(l)}(\vec{s}_0)$ numerically. Therefore, we interpret the structure of zeros and ones in $\tilde{w}^{(l)}(\vec{s}_0)$ as fixing this $\mathrm{GL}(M)$ freedom in the phase-space independent basis. Secondly, many of the entries of the evaluation $\tilde{w}_{jk}^{(l)}(\vec{s}_0)$ are either zero, or identical. It is natural to interpret these zeros/equalities as phase-space independent. Altogether, we are able to write an ansatz for the $\mathrm{GL}(M)$ fixed basis as

$$\tilde{w}_{jk}^{(l)} = \tilde{w}_{jk}^{(l)[0]} + \sum_{m=1}^{e} y_m(\vec{s})\tilde{w}_{jk}^{(l)[m]}, \tag{5.19}$$

where $e$ is the number of distinct, non-zero (and not equal to 1) entries of $\tilde{w}_{jk}^{(l)}(\vec{s}_0)$ and the entries of each $\tilde{w}_{jk}^{(l)[m]}$ are either zero or one. Inserting the basis ansatz eq. (5.19) into the power-counting constraints eq. (5.18), we end up with a linear system for the $y(\vec{s})$ as

$$\sum_{m=1}^{e} A_{nm}y_m(\vec{s}) = b_n, \tag{5.20}$$

where the inhomogeneous term on the right-hand side is a consequence of the inhomogeneous term, $w^{(l)[0]}$, of eq. (5.19). In this way, the $y_m$ can be fixed by solving a simpler linear system than eq. (5.18). Importantly, $e$ is often very small and so this represents an important speed up.

In summary, given a generating set of CSyz(Γ), we construct a basis of a space of surface terms $\mathrm{CSyzSurface}^R(\Gamma, \vec{\nu}) \subset \mathrm{Surface}^R(\Gamma, \vec{\nu})$. The construction is performed for a given numerical phase-space point, and optimized by structures learned from an initial evaluation.

### 5.1.3 Summary of approach

Let us now summarize our approach for critical surface term generation. We use critical syzygies to generate a set of surface terms within gluonic power counting associated to a given diagram Γ that are linearly independent on the maximal cut associated to Γ. By collecting these surface terms for all diagrams Γ, one then has the necessary ingredients for integral reduction without raising propagator powers. The resulting surface terms are implicitly stated

JHEP02(2026)004

as a set of analytic linear systems that can be solved numerically on a given phase-space point. The approach makes use of the Baikov representation, taking the Baikov polynomial as input. In principle, this can be the Baikov polynomial of the top topology, or the Baikov polynomial associated to the graph itself. Our approach is composed of three major steps.

1. Working on a numerical phase-space point, we compute the saturation index $\mu$ of the $J^{\Gamma}_{\text{syz}}$ with respect to the Baikov polynomial to check if it is 1. We compute the dimension of $U^{\Gamma}_{\text{crit}[\log(B)]}$ to check if it is zero. If either of these checks fail, we abort.[11]

2. We construct a generating set of CSyz($\Gamma$) following the discussion of section 5.1.1. We fix an initial polynomial degree bound for the ansatz, $\vec{N}$, typically $N_j = 2$. We construct a tentative generating set with this degree bound and check if it generates CSyz($\Gamma$) via eq. (5.8). If not, we raise the degree bounds in an ad-hoc manner until we successfully find a sufficiently high degree bound. The resulting generating set is presented as the solution to an analytic linear equation system, eq. (5.12), which we optimize following the discussion in the latter part of section 5.1.1.

3. Given this generating set of CSyz($\Gamma$), we construct a basis of CSyzSurface$^R(\Gamma, \vec{\nu})$ as discussed in section 5.1.2. To maintain compact results, we employ two possible strategies to construct these surface terms. We first construct monomial multiples of our generating set. If such syzygies do not span the power counting space, we construct appropriate linear combinations via linear algebra. If this results in an incomplete set of surface terms, we return to step 2. We increase the degree bounds $\vec{N}$ and construct a new, larger generating set of CSyz($\Gamma$). We repeat this iteratively until an appropriate $\vec{N}$ has been found such that a complete set of surface terms is produced. The resulting basis of power-counting compatible surface terms is then either implicitly stated as an analytic linear system, eq. (5.18), or as monomial multiples of CSyz($\Gamma$) generators.

The output of this procedure is two fold. First, there is an implicit representation of a generating set of CSyz($\Gamma$) as a compact, analytic linear system in eq. (5.12). Second, the basis of surface terms is either presented implicitly as a further compact, analytic linear system, eq. (5.18) or as explicit products of monomials in Baikov variables and CSyz($\Gamma$) generators. The linear systems can then be solved to produce the necessary surface terms. In practice, it is most fruitful to solve these systems numerically, as we do in section 5.2.

Let us make a few comments on our approach. Firstly, we do not guarantee that it produces a basis that spans CSyzSurface$^R(\Gamma, \vec{\nu})$. That is, we do not guarantee that the isomorphism of eq. (5.3) holds. Nevertheless, we find in practical applications that it does. Secondly, in step 3, we see that we cannot use an arbitrary generating set of CSyz($\Gamma$) to construct a set of critical surface terms within power counting by taking linear combinations. For this reason, we allow ourselves to return to step 2 and increase the degree bounds.

### 5.2 Application to planar contributions to $pp \to t\bar{t}H$ at two loops

In order to show that critical syzygies are a useful tool in practical application to scattering processes, we apply our approach to the two-loop leading-color contributions to the $pp \to t\bar{t}H$

[11] Study of the higher dimensional case is beyond the scope of this paper and left for future work. We discuss an ad-hoc solution to a single $\mu \neq 1$ case in section 5.2.

JHEP02(2026)004

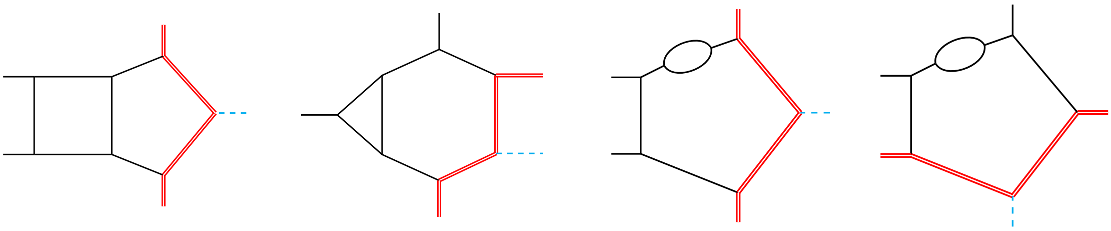

**Figure 3.** Four top sectors of leading color ttH production with light quark loop. Red lines denote the top quark, the blue dashed line denotes the Higgs boson. Black lines are massless particles (gluons or light quarks).

process. Importantly, this allows us to study, in a physically relevant case, the strength of the assumption that the saturation index, $\mu$, is 1 and the critical locus of the logarithm of the Baikov polynomial, $U^{\Gamma}_{\text{crit}(\log[B])}$, is a finite set of points.

We begin by applying the approach in section 5.1.3 to generate a power-counting-compatible basis of surface terms for all diagrams in leading-color light-quark-loop contributions in $t\bar{t}H$ production. With a view to implementing these surface terms in the `Caravel` framework, for each graph $\Gamma$ we work with the Baikov polynomial associated to this graph (and not the top topology) as the remaining class of surface terms are known analytically [29]. Diagrams depicting the four top-sectors are shown in figure 3. We find that there are 123 non-factorizable subsectors which are inequivalent under relabelling of external legs. As our representation of surface terms is analytic, it is sufficient to analyze only these inequivalent sectors. We compute the saturation index $\mu$ and the dimension of the critical locus of the cut Baikov polynomial, using the computer algebra system `Singular`. We perform the computation of both quantities on a numerical, finite-field phase-space point, which leads to a negligible computation time.

Of the 123 inequivalent topologies, 122 give rise to a saturation index of $\mu = 1$ and a critical locus of $\log(B)$ on the maximal cut which is a finite set of points. We consider these first. Constructing the surface terms with our approach for each sector requires solving linear equation system in eq. (5.12). In practice, we find that the largest linear equation system is of side-length 400. In order to find a basis of surface terms within power counting, we must choose a strategy for solving the power-counting constraints eq. (5.18). In most cases, we find it sufficient to use the first strategy. Nevertheless, there are 9 sectors for which we apply the second strategy. In this case, after optimization, the most complicated linear system that we must solve for the unknowns $y_m$ of eq. (5.19) contains 80 unknowns. Having constructed the complete set of surface terms, we see that all generating sets for the $\text{CSyz}(\Gamma)$ can be constructed with degree bounds satisfying $N_j \leq 3$.

The single topology that has a saturation index of $\mu = 2$ is depicted in figure 4. We note that the critical locus of the logarithm of the Baikov polynomial is still a finite set of points. To compute the set of surface terms in this case, we take a two step approach. First, we use the approach of section 5.1.3 to compute as many surface terms as possible. In practice, we find that we miss only one surface term. We then turn to the analysis of section 3.5 to construct the remaining surface term. We first compute the saturation index $\overline{\Delta}$ and find that it is 2, telling us that this construction does indeed produce a single new surface term in

JHEP02(2026)004

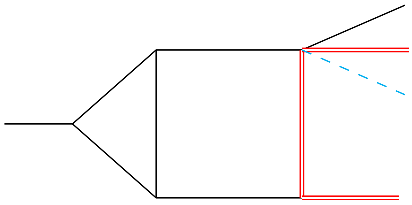

**Figure 4.** $t\bar{t}H$ topology for which the saturation index of $J^{\Gamma}_{\rm syz}$ with respect to the Baikov polynomial is not 1. In this case, to recover the full set of surface terms, we allow for dimension shifted seed integrals. Black lines represent massless particles (i.e. gluons/light quarks), red lines represent the top quark and the blue dashed line represents the Higgs.

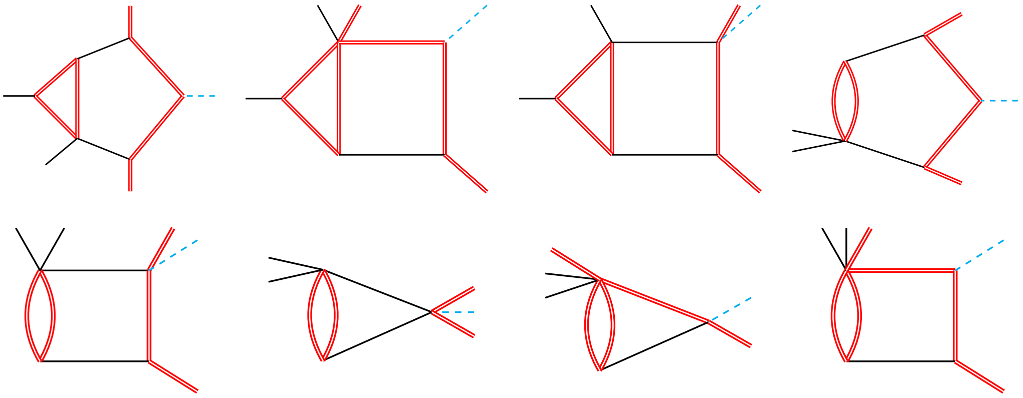

**Figure 5.** Collection of non-factorizable topologies from planar massive quark loop contributions to $pp \to t\bar{t}H$ for which the critical locus of $\log(B)$ is not isolated. Red lines represent massive top quarks, black lines represent massless particles (gluons) and the blue line represents the Higgs. All integral topologies that contribute to $pp \to t\bar{t}H$ which have a non-isolated critical locus are either permutations of those displayed, or are factorizable.

JHEP02(2026)004

the large $\epsilon$, on-shell limit. To explicitly construct the surface term, we then solve eqs. (3.41) and (3.42) by using a polynomial ansatz, successfully recovering the remaining surface term and therefore constructing the full set. We note that, due to the high degree of the Baikov polynomial, this is quite computationally demanding. Nevertheless, the resulting surface term is quite simple. It is interesting to also analyze this topology with the traditional Laporta approach. We use LiteRed [59] to generate IBP relations using only single propagator powers as seeds and find that a single relation is also missing. However, if one allows for higher degree powers of the propagators in seed integrals, then a complete reduction is observed. This suggests that it would be interesting to study how to interpret seeds with raised propagator powers in a critical-syzygy framework, a question we leave to future work.

In order to check the validity of the surface terms that we produce, we implement the analytic systems of linear equations that define the surface terms into `Caravel` [32], which is able to use such systems to perform numerical reductions of tensor integrals. We then use `Caravel` to perform numerical reductions of tensor integrals within power counting and check the validity of our implementation with `FIRE 6.5` [12] at a series of randomly chosen numerical phase-space points.

Having considered the light-fermion-loop contributions to $pp \to t\bar{t}H$, we next make an analysis of the pentabox topology that contributes to the closed-top-loop contributions. Here, we find a number of topologies for which the critical locus of the logarithm of the Baikov polynomial is not a finite set of points. Many of these topologies are factorizable, containing the one-loop integral of figure 1 as a factor, so this observation is natural. Nevertheless, many of these integrals are not factorizable, and we depict them in figure 5. For these topologies, critical syzygies as studied in this paper are insufficient to perform a complete reduction to master integrals, and we leave such a study to further work. Interestingly, for each topology depicted in figure 5, when considered in the Baikov representation associated to corresponding graph, the critical locus on the maximal cut is only one-dimensional. This unexpected simplicity hints that extension of the critical syzygy formalism to cover these cases may be within reach.

## 6 Summary and outlook

In this work, we have uncovered a new mathematical structure hidden within the linear relations exhibited by Feynman integrals. Working in the syzygy approach for constructing relations between Feynman integrals and motivated by recent advances in intersection theory, we considered how the numerators of integral relations, or "surface terms" behave in the limit where the dimensional regulator, $\epsilon$, is taken to be large. We showed how surface terms in the large-$\epsilon$ on-shell limit, must vanish on critical points of the logarithm of the Baikov polynomial. Moreover, we showed how this statement can be interpreted in the algebro-geometric language of ideals, and how the ideals that arise are connected to the Lee-Pomeransky approach for counting the number of master integrals. This connection then motivated us to define a special class of syzygies, which we dubbed "critical syzygies". Strikingly, while critical syzygies are effectively a subset of the full syzygy module, for cases where the critical locus of the Baikov polynomial is a finite set of points, we argued that they can be used to construct the full set of surface terms in the large-$\epsilon$ limit.

In order to understand the practical construction of critical syzygies for loop amplitude calculations, we made a number of studies at the one- and two-loop level. We first discussed how critical syzygies arise at one loop, providing an alternative construction of OPP-like integrand bases. We then moved to consider two-loop approaches, where we presented a computational approach to construct critical syzygies. We used this approach to study the two-loop example of planar Feynman integrals for contributions to $pp \to t\bar{t}H$ production, directly showing the applicability of critical syzygies to the light-fermion-loop case. Interestingly, this allowed us to identify a case where careful study of the multiplicity structure of the syzygies becomes important to obtain a complete reduction to master integrals.

There are a number of further important avenues for work within the critical syzygy approach. Firstly, critical syzygies are insufficient to perform IBP reduction in cases where the critical locus of the cut Baikov polynomial is not a finite set of points. A natural extension of our work would be to understand if the critical syzygy framework could be extended to cover such cases. Moreover, our study in section 5.2 of the integral topology relevant to $t\bar{t}H$ production where the multiplicity structure plays an important role suggests that it would be useful to understand higher propagator seeding in a critical syzygy framework. Finally, the

JHEP02(2026)004

geometrical connection between critical syzygies and critical/singular points of the Baikov polynomial motivates further work into constructing analytical critical syzygy solutions.

## Acknowledgments

We thank Giulio Gambuti, Harald Ita, Pavel Novichkov and Vasily Sotnikov for insightful discussions. We thank Harald Ita and Pavel Novichkov for comments on the draft. The work of Qian Song was supported by the European Research Council (ERC) under the European Union's Horizon Europe research and innovation program grant agreement 101078449 (ERC Starting Grant MultiScaleAmp). Views and opinions expressed are however those of the authors only and do not necessarily reflect those of the European Union or the European Research Council Executive Agency. Neither the European Union nor the granting authority can be held responsible for them.

## A Extended splitting lemma

Here we prove the lemma necessary to decompose $J^{\Gamma}_{\text{syz}}$ in section 3.2. This lemma can be regarded as an extended version of the splitting lemma described in [18, section B.3].

**Lemma A.1.** *Let $R$ be a polynomial ring, $J, K$ be ideals of $R$ and $b$ be an element of $R$ such that $Kb \subset J$. In this case one has that*

$$J = (J + K) \cap (J + b^{\mu}), \tag{A.1}$$

*where $\mu$ is the saturation index of $b$ with respect to $J$.*

*Proof.* It is clear that $J \subseteq (J + K) \cap (J + b^{\mu})$ as the intersection is of two sets which each contain $J$. Hence, if we have the reverse (non-proper) inclusion, then we have equality. Let us consider an element of the intersection. We shall prove that it is a member of $J$, which will therefore prove (A.1).

Let us name the element in question $c$. By definition we can write that

$$c = j_1 + k = j_2 + tb^{\mu}, \tag{A.2}$$

where $j_i \in J$, $k \in K$ and $t \in R$. We aim to prove that $tb^{\mu} \in J$. To do this, we shall consider

$$tb^{\mu+1} = j_1 b + kb - j_2 b. \tag{A.3}$$

Manifestly the right hand side is a sum of three elements of $J$, and as $J$ is an ideal this implies that $tb^{\mu+1}$ is also an element of $J$. By the definition of an ideal quotient, we have that this means that $t \in J : b^{\mu+1}$. However, as $\mu$ is chosen to be the saturation index of $b$, we have that $J : b^{\mu+1} = J : b^{\mu}$, which implies that $tb^{\mu} \in J$. Looking back to the definition of $c$, it is clear that $c \in J$ and therefore the equality in (A.1) is proven. □

JHEP02(2026)004

**Data Availability Statement.** This article has no associated data or the data will not be deposited.

**Code Availability Statement.** This article has no associated code or the code will not be deposited.

**Open Access.** This article is distributed under the terms of the Creative Commons Attribution License (CC-BY4.0), which permits any use, distribution and reproduction in any medium, provided the original author(s) and source are credited.
